# Supplementary material for: Endogenous topoisomerase II-mediated DNA breaks drive thymic cancer predisposition linked to ATM deficiency
Source: Nat Commun. 2020 Feb 14;11:910. doi: 10.1038/s41467-020-14638-w (PMC7021672; doi:10.1038/s41467-020-14638-w)
Supplement: Supplementary file 1 — Supplementary Information [file 41467_2020_14638_MOESM1_ESM.pdf]

A

Genotype	Observed	Expected
Tdp2$^{+/+}$*Atm*$^{+/+}$	24	32
Tdp2$^{+/+}$*Atm*$^{+/-}$	70	75
Tdp2$^{+/+}$*Atm*$^{-/-}$	27	32
Tdp2$^{+/-}$ *Atm*$^{+/+}$	58	64
Tdp2$^{+/-}$ *Atm*$^{+/-}$	152	130
Tdp2$^{+/-}$ *Atm*$^{-/-}$	69	65
Tdp2$^{-/-}$ *Atm*$^{+/+}$	27	32
Tdp2$^{-/-}$ *Atm*$^{+/-}$	60	65
Tdp2$^{-/-}$ *Atm*$^{-/-}$	31	32
Total	518	

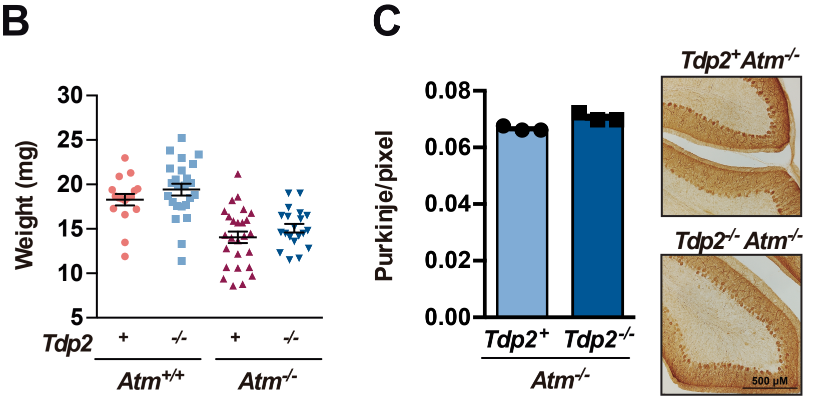

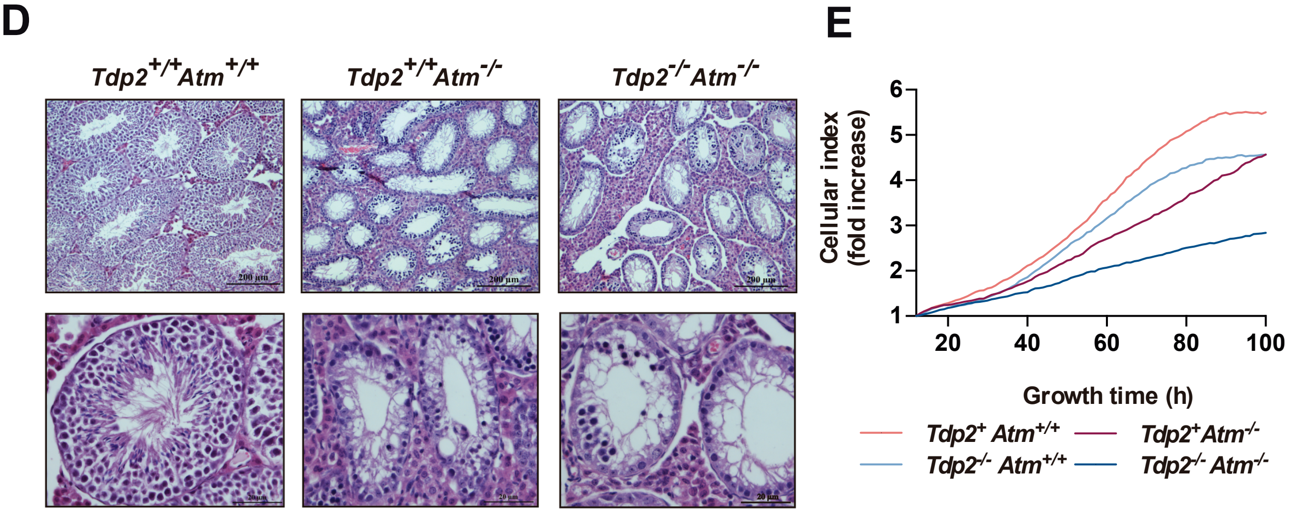

Supplementary Figure 1. TDP2 deficiency does not impact development of *Atm*$^{-/-}$ mice despite defects in cellular growth. (A) Observed genotypes of the progeny of crosses between *Tdp2 Atm* double-heterozygote mice and expected numbers according to mendelian proportions. **(B)** Weight of mice of the indicated genotypes 4 weeks after birth. Mean +/- s.e.m. is shown. n=17 (*Tdp2*$^{+/+}$ *Atm*$^{+/+}$*)*, n=24 (*Tdp2*$^{-/-}$ *Atm*$^{+/+}$*)*, n=26 (*Tdp2*$^{+/+}$ *Atm*$^{-/-}$), n=20 (*Tdp2*$^{-/-}$ *Atm*$^{-/}$). **(C)** Purkinje cellular density observed by calbindin immunohistochemistry on sagittal vibratome sections of cerebella obtained from *Tdp2*$^{+}$*Atm*$^{-/-}$ and *Tdp2*$^{-/-}$*Atm*$^{-/-}$ 8 weeks old mice. Mean +/- s.e.m. of three independent individuals per genotype were analysed (n=3). Quantification (left) and representative images (right) are shown. **(D)** Sections of testes from adult *wild-type*, *Atm*$^{-/-}$ and *Tdp2*$^{-/-}$ *Atm*$^{-/-}$ mice stained with Haematoxylin-Eosin. Two independent mice were analysed per genotype with similar results. **(E)** Cellular growth index along 3 days in the indicated genotypes of primary MEFs.

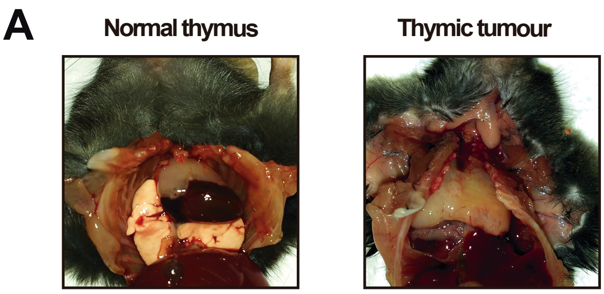

C

	Tdp2⁺ *Atm*⁻/⁻	*Tdp2*⁻/⁻ *Atm*⁻/⁻
**CD4-CD8-**	1.98 ± 0.90	1.74 ± 0.54
**CD4+CD8-**	3.36 ± 1.54	3.20 ± 0.59
**CD4-CD8+**	1.22 ± 0.22	1.30 ± 0.40
**CD4+CD8+**	93.44 ± 0.22	93.76 ± 2.48

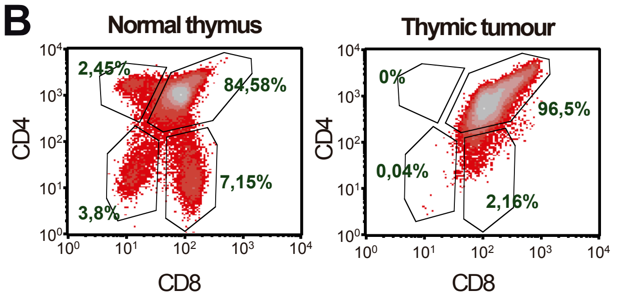

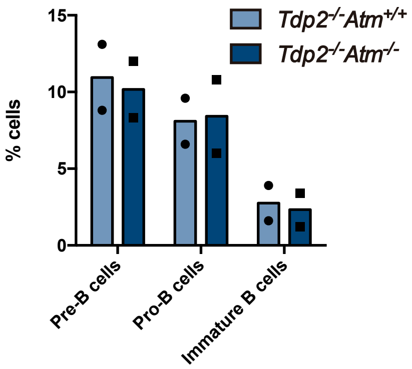

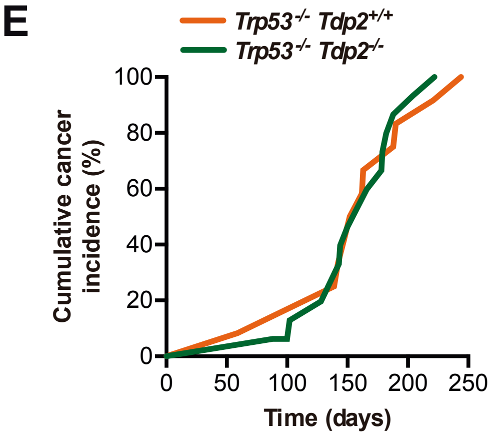

Supplementary Figure 2. Phenotypic characterization of thymic tumours in *Atm*⁻/⁻ and *Tdp2*⁻/⁻ *Atm*⁻/⁻ mice. (A) Representative distribution of CD4 and CD8 markers in thymocytes and **(B)** macroscopic image of the thymus in an 8-week old *Tdp2*⁻/⁻ *Atm*⁻/⁻ healthy mouse (normal thymus, left) and one suffering a thymic tumour (right). **(C)** Distribution (percentage) of double negative (CD4-CD8-), single positive (CD4+CD8- and CD4-CD8+) and double positive (CD4+CD8+) thymocytes in 8-week old mice of the indicated genotype. At least 3 mice of each genotype were analysed. *Tdp2*⁺ indicates both *Tdp2*⁺/⁺ and *Tdp2*⁺/⁻. **(D)** Distribution (percentage) of Pro-b cell (CD43+ B220+), Pre-B cell (CD43− B220^{low}) immature B cell (CD43− B220^{high}) in 5-weeks old mice of the indicated genotypes (n=2) analysed by FACs. Cells were gated by size and complexity prior classification by levels of CD43 and B220 markers. **(E)** Cumulative occurrence of thymic lymphoma in the indicated *Tdp2* and *Trp53* genotypes (n=12 and n=16 for *Tdp2*⁺/⁺ and *Tdp2*⁻/⁻ mice respectively).

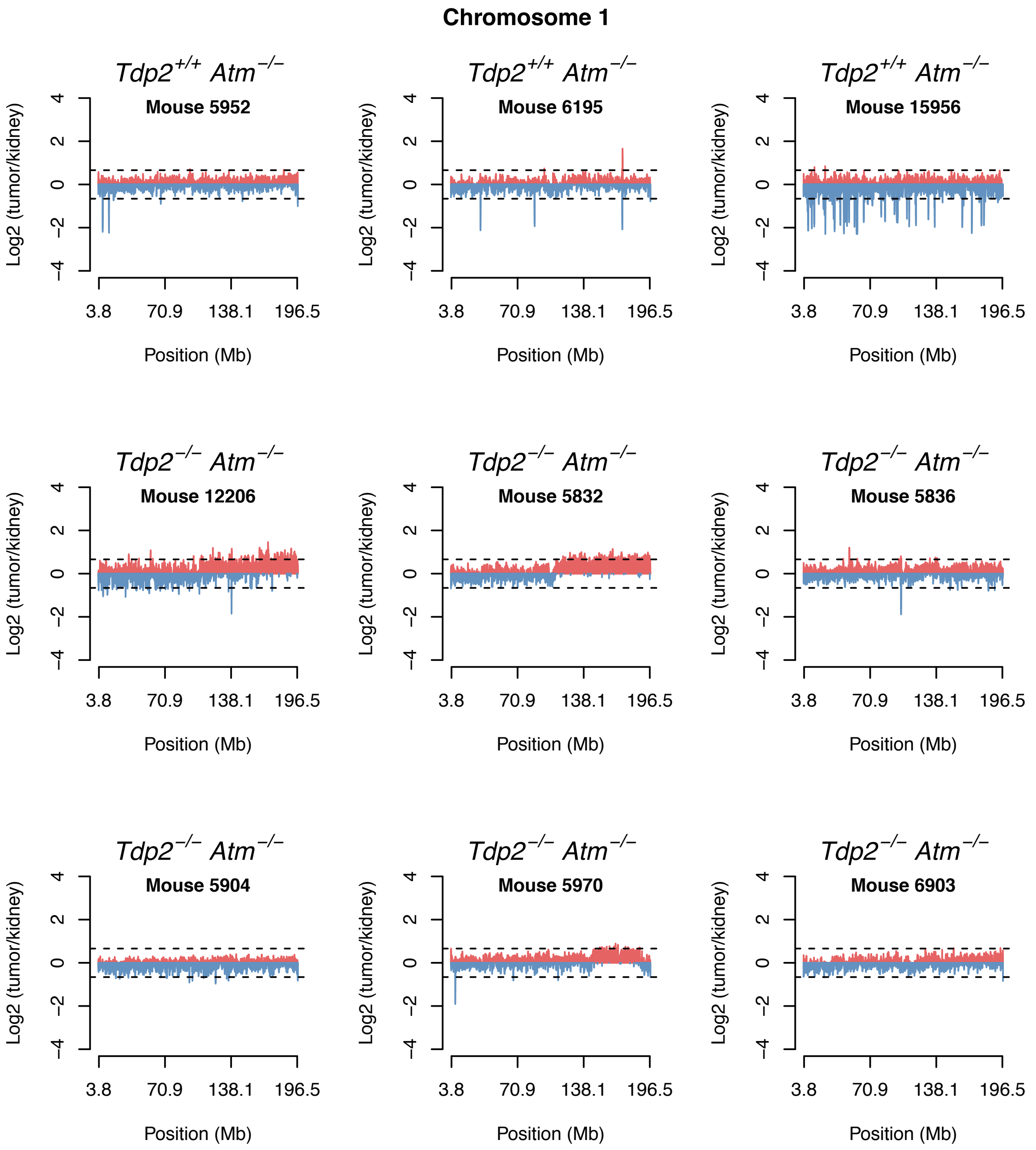

Supplementary Figure 3. Genomic characterization of thymic tumours in *Atm*$^{-/-}$ and *Tdp2*$^{-/-}$ *Atm*$^{-/-}$ mice. Individual CGH analysis in each chromosome of *Tdp2*$^{+/+}$*Atm*$^{-/-}$ and *Tdp2*$^{-/-}$*Atm*$^{-/-}$ thymic lymphomas. The ID number for each mouse is indicated. DNA from each tumour sample was hybridized and analysed using kidney DNA from the same mouse as a control. Amplification (red) or deletion (blue) score (Log2 tumour/kidney ratio) is shown. Significant copy number variations are defined by -0.66>Log2 tumour/kidney>0.66 (dashed lines). The location of relevant loci in *Atm*$^{-/-}$ thymic tumours is indicated.

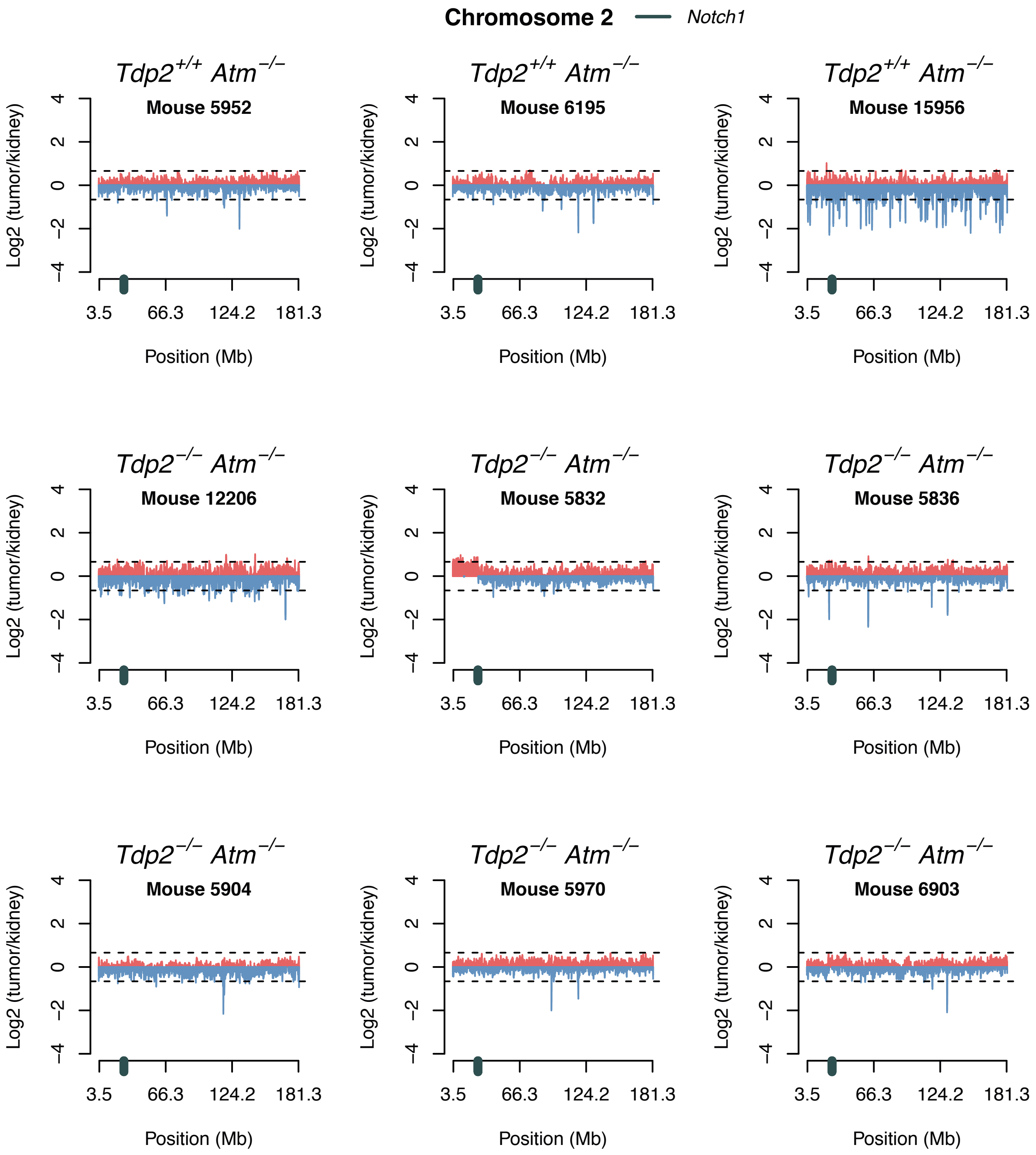
Chromosome 2
Notch1
Tdp2+/+ Atm−/−
Mouse 5952
Tdp2+/+ Atm−/−
Mouse 6195
Tdp2+/+ Atm−/−
Mouse 15956
Tdp2−/− Atm−/−
Mouse 12206
Tdp2−/− Atm−/−
Mouse 5832
Tdp2−/− Atm−/−
Mouse 5836
Tdp2−/− Atm−/−
Mouse 5904
Tdp2−/− Atm−/−
Mouse 5970
Tdp2−/− Atm−/−
Mouse 6903
Log2 (tumor/kidney)
−4
−2
0
2
4
3.5
66.3
124.2
181.3
Position (Mb)

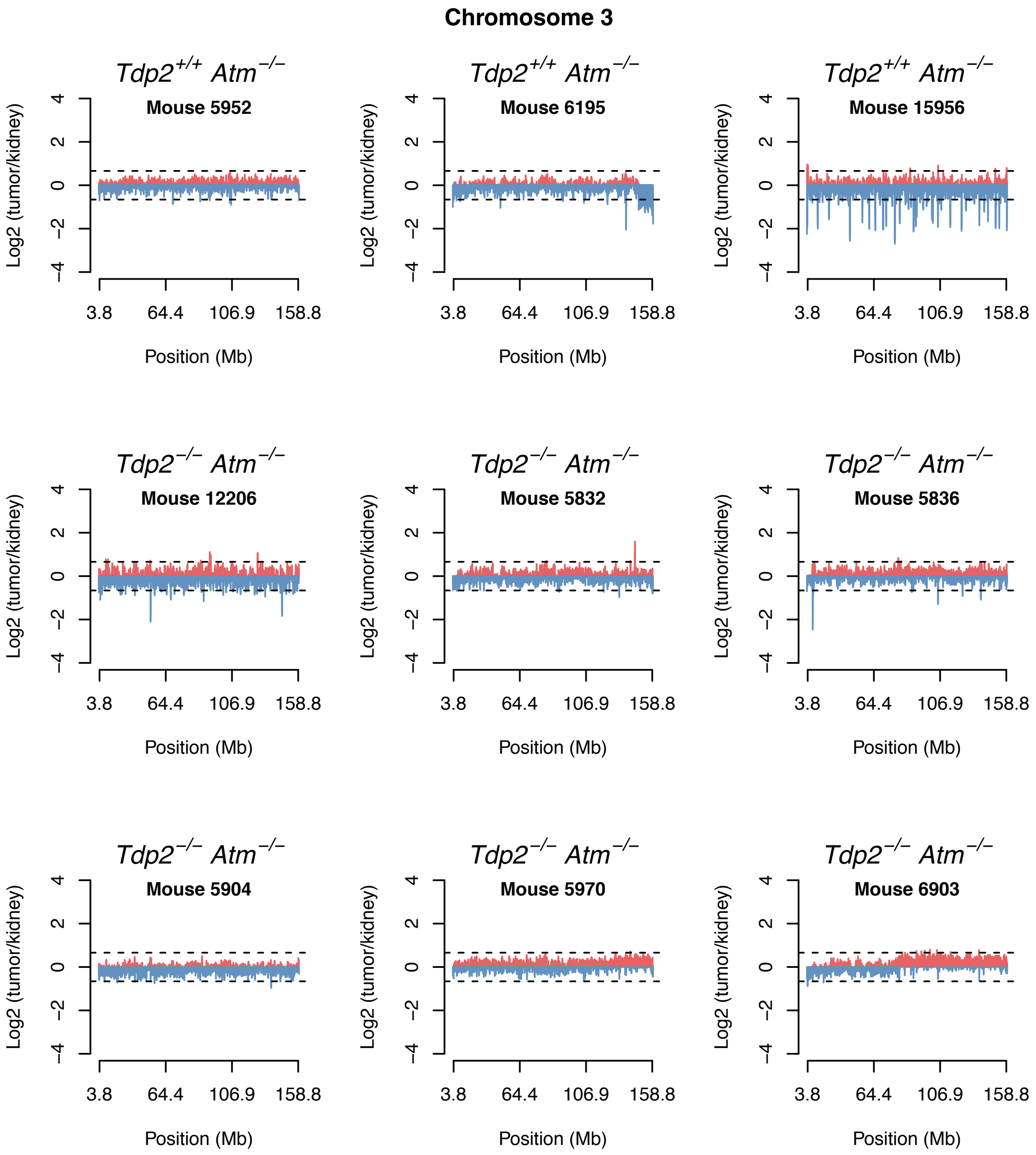
Chromosome 3
Tdp2+/+ Atm−/−
Mouse 5952
Tdp2+/+ Atm−/−
Mouse 6195
Tdp2+/+ Atm−/−
Mouse 15956
Tdp2−/− Atm−/−
Mouse 12206
Tdp2−/− Atm−/−
Mouse 5832
Tdp2−/− Atm−/−
Mouse 5836
Tdp2−/− Atm−/−
Mouse 5904
Tdp2−/− Atm−/−
Mouse 5970
Tdp2−/− Atm−/−
Mouse 6903
Log2 (tumor/kidney)
−4 −2 0 2 4
3.8 64.4 106.9 158.8
Position (Mb)

Chromosome 4

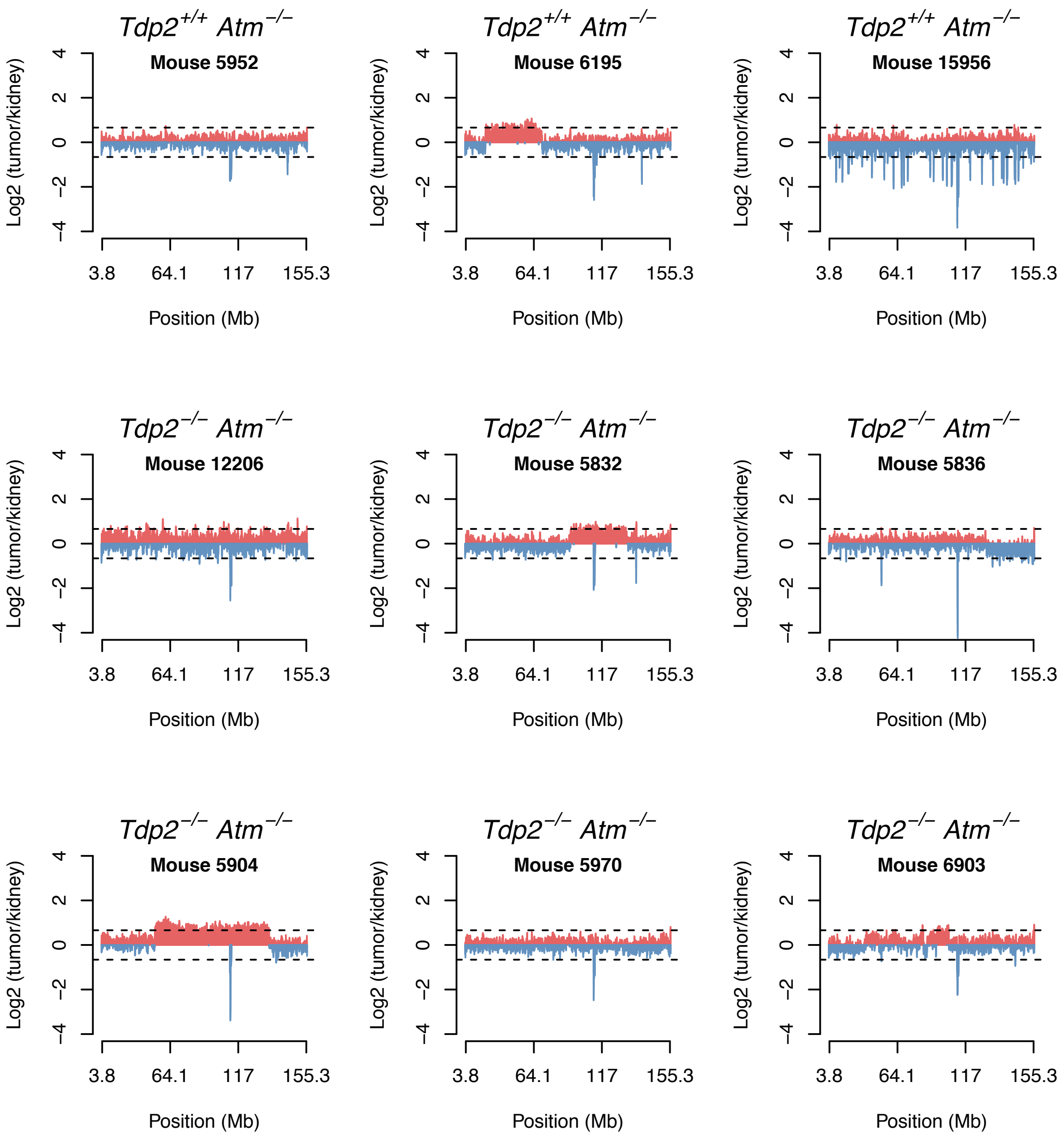

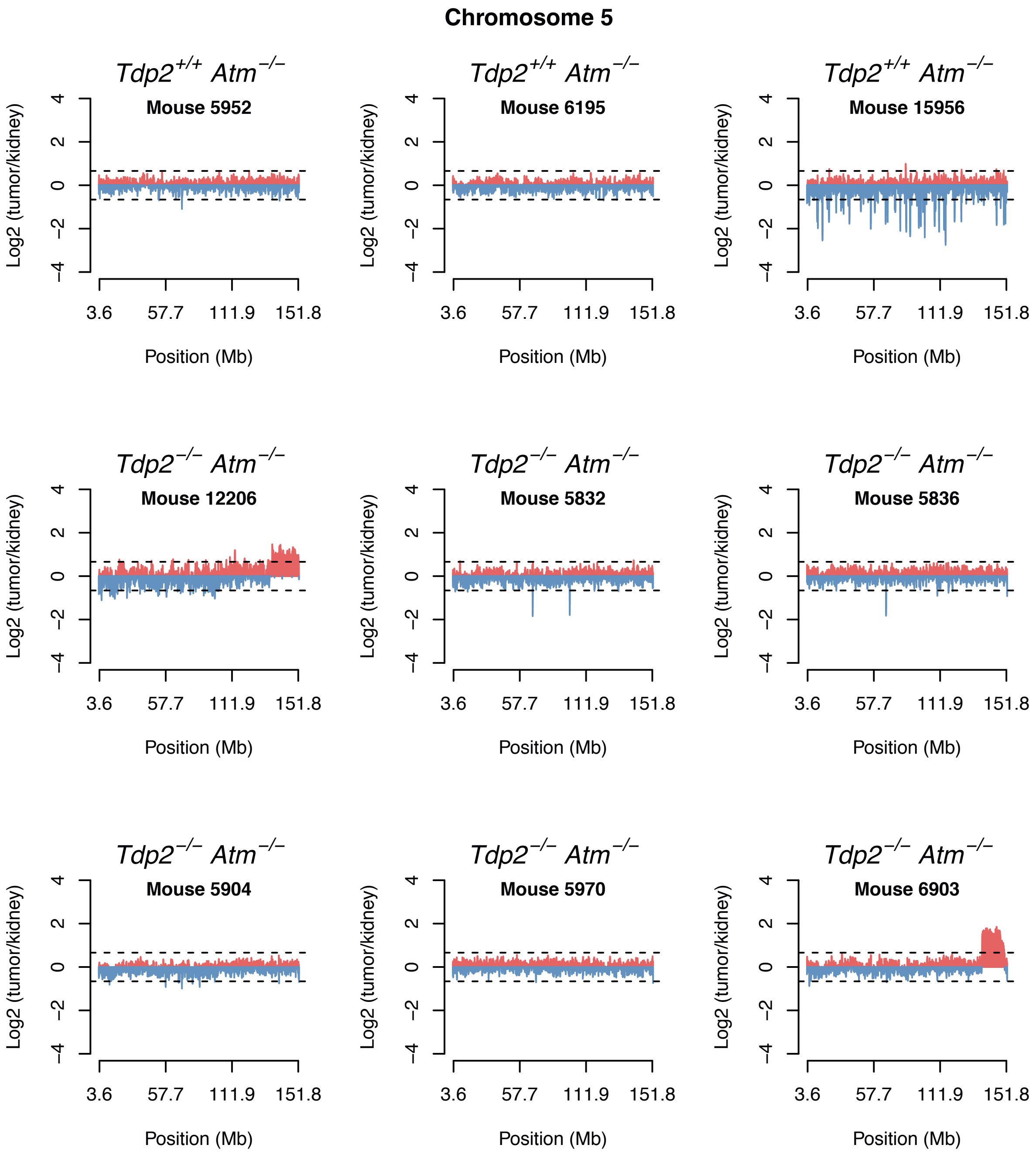
Chromosome 5
Tdp2+/+ Atm−/−
Mouse 5952
Tdp2+/+ Atm−/−
Mouse 6195
Tdp2+/+ Atm−/−
Mouse 15956
Tdp2−/− Atm−/−
Mouse 12206
Tdp2−/− Atm−/−
Mouse 5832
Tdp2−/− Atm−/−
Mouse 5836
Tdp2−/− Atm−/−
Mouse 5904
Tdp2−/− Atm−/−
Mouse 5970
Tdp2−/− Atm−/−
Mouse 6903
Log2 (tumor/kidney)
4
2
0
−2
−4
3.6
57.7
111.9
151.8
Position (Mb)

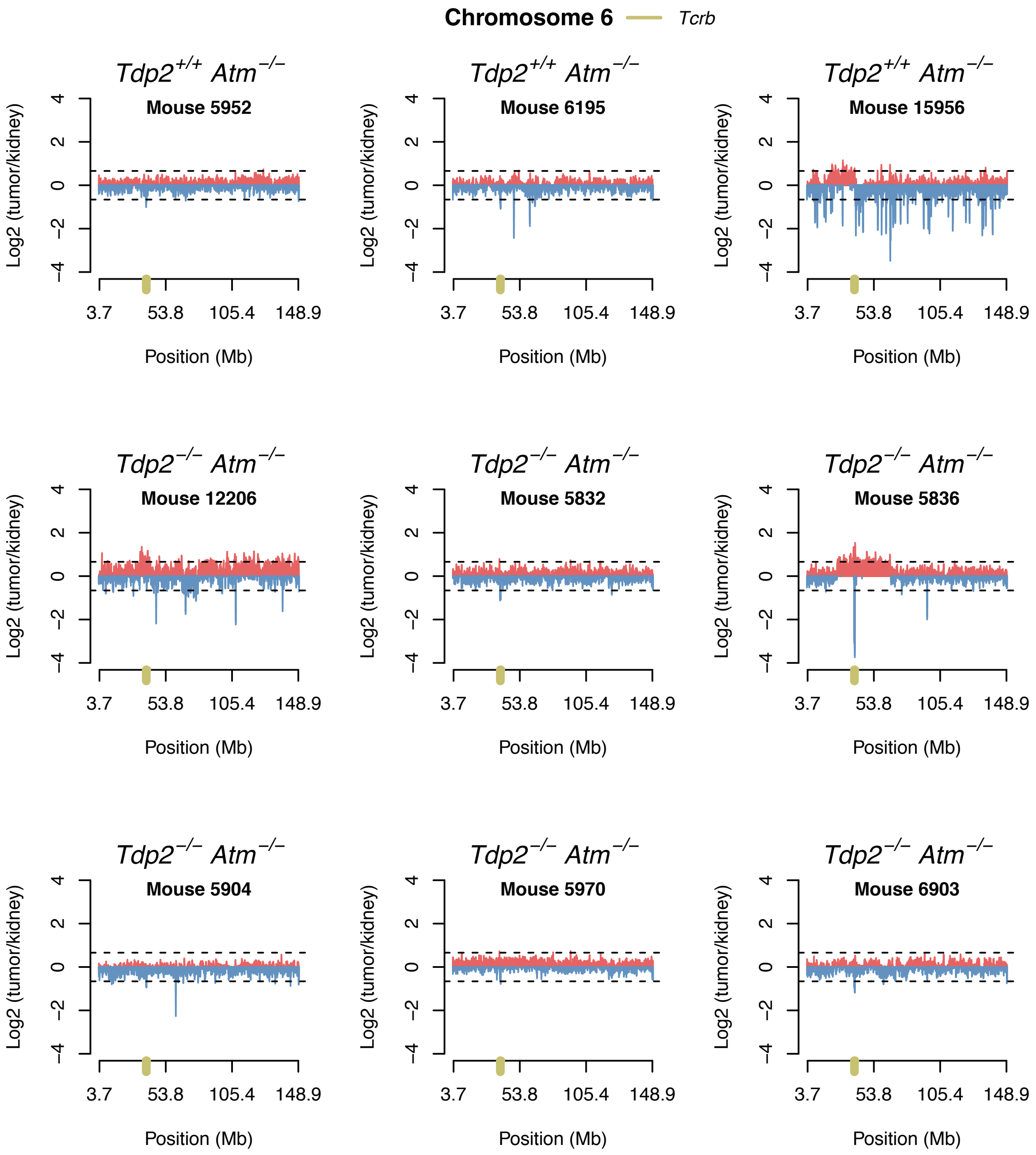

Chromosome 6
Tcrb
Tdp2+/+ Atm−/−
Mouse 5952
Mouse 6195
Mouse 15956
Tdp2−/− Atm−/−
Mouse 12206
Mouse 5832
Mouse 5836
Mouse 5904
Mouse 5970
Mouse 6903
Log2 (tumor/kidney)
4
2
0
−2
−4
3.7
53.8
105.4
148.9
Position (Mb)

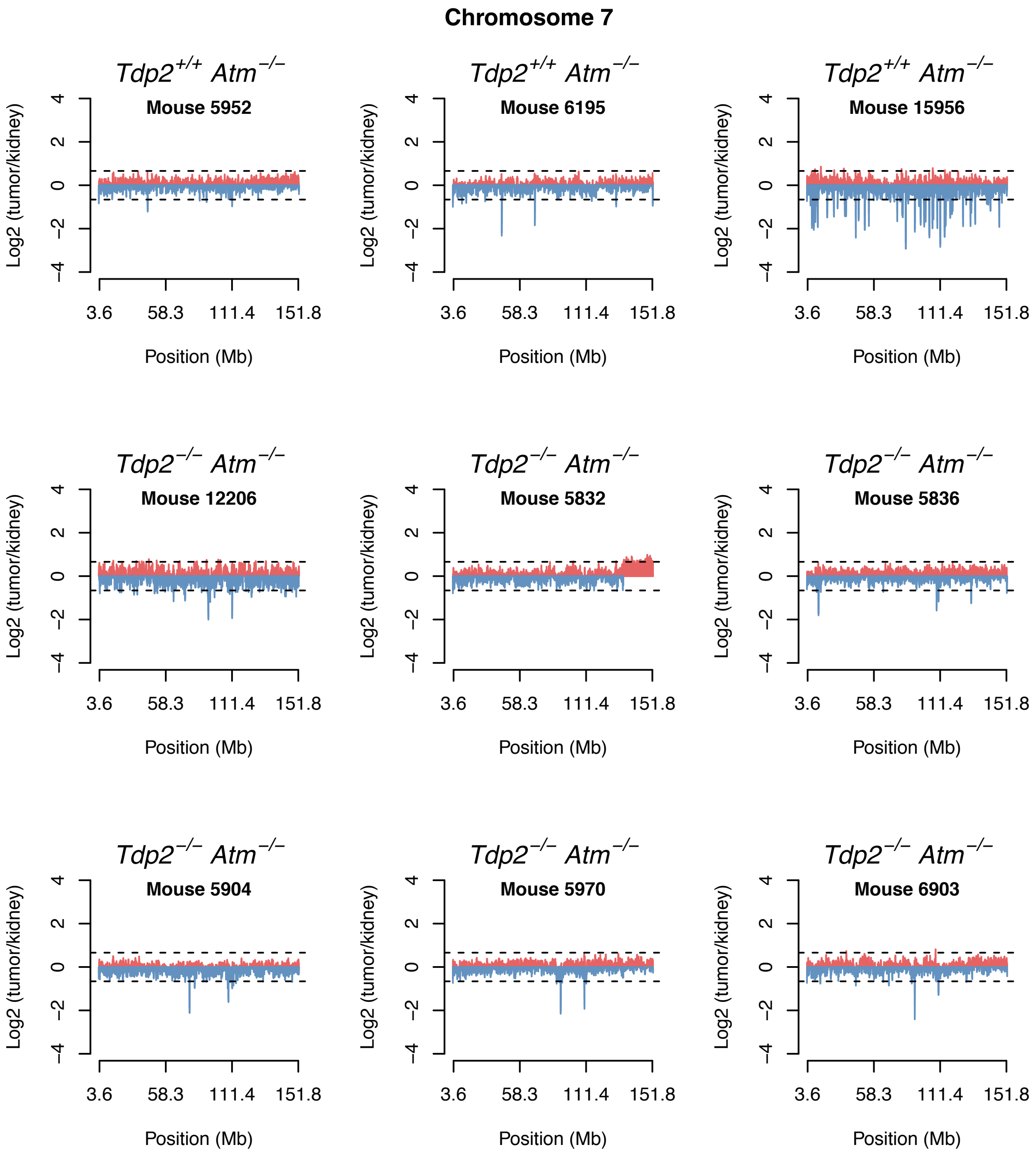
Chromosome 7
Tdp2+/+ Atm−/−
Mouse 5952
Mouse 6195
Mouse 15956
Tdp2−/− Atm−/−
Mouse 12206
Mouse 5832
Mouse 5836
Mouse 5904
Mouse 5970
Mouse 6903
Log2 (tumor/kidney)
4
2
0
−2
−4
3.6
58.3
111.4
151.8
Position (Mb)

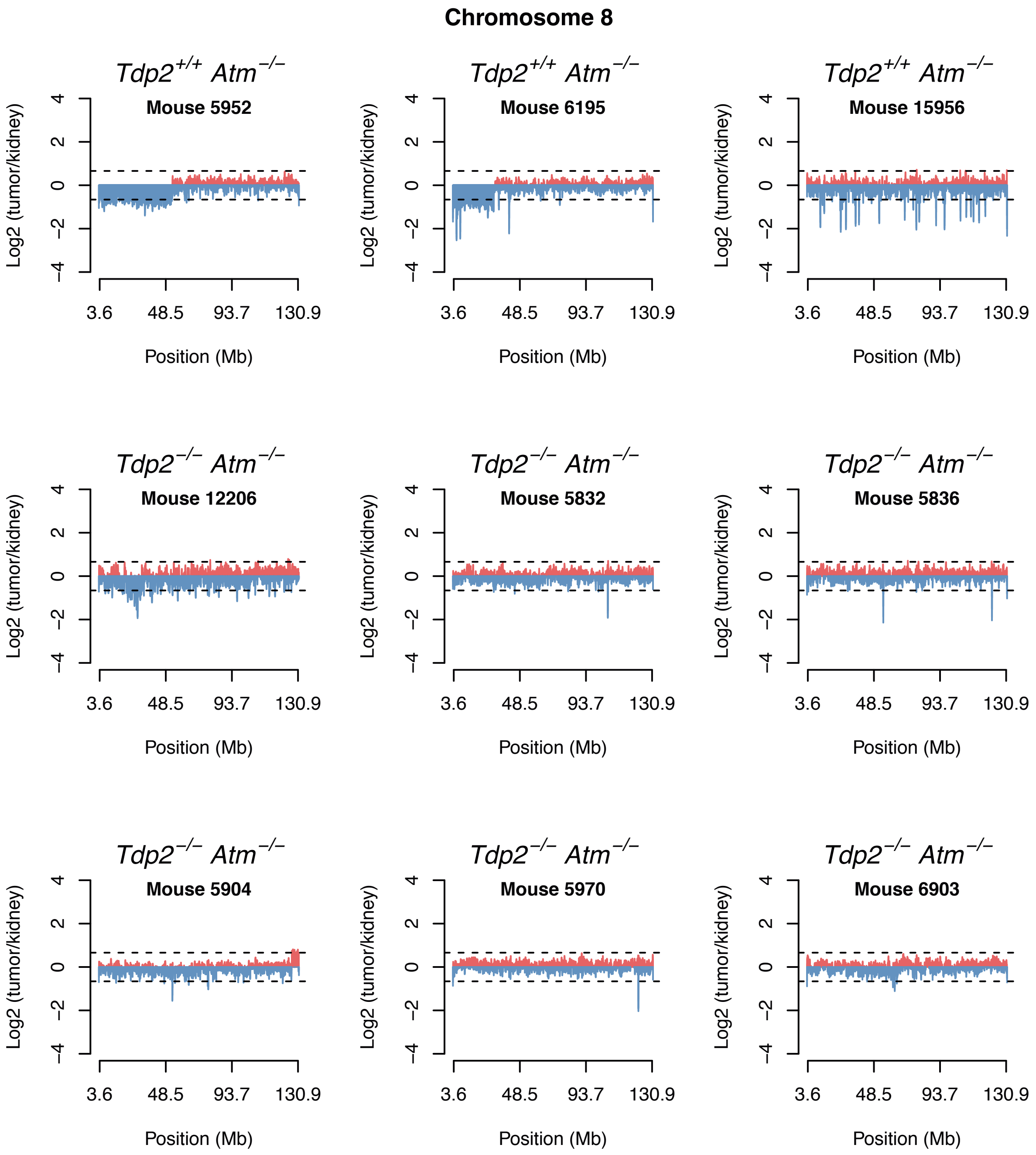
Chromosome 8
Tdp2+/+ Atm−/−
Mouse 5952
Tdp2+/+ Atm−/−
Mouse 6195
Tdp2+/+ Atm−/−
Mouse 15956
Tdp2−/− Atm−/−
Mouse 12206
Tdp2−/− Atm−/−
Mouse 5832
Tdp2−/− Atm−/−
Mouse 5836
Tdp2−/− Atm−/−
Mouse 5904
Tdp2−/− Atm−/−
Mouse 5970
Tdp2−/− Atm−/−
Mouse 6903
Log2 (tumor/kidney)
−4
−2
0
2
4
3.6
48.5
93.7
130.9
Position (Mb)

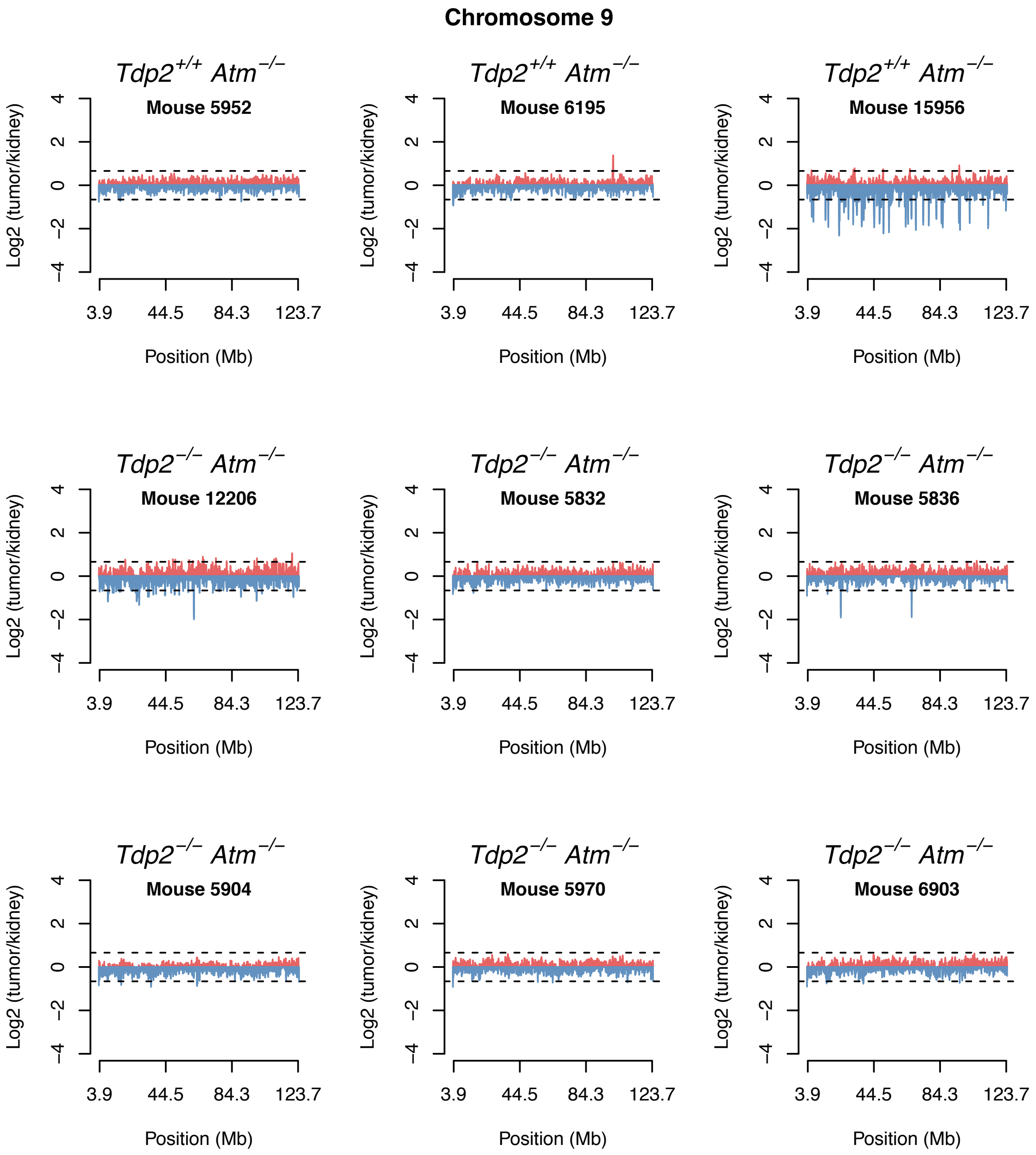
Chromosome 9
Tdp2+/+ Atm−/−
Mouse 5952
Mouse 6195
Mouse 15956
Tdp2−/− Atm−/−
Mouse 12206
Mouse 5832
Mouse 5836
Mouse 5904
Mouse 5970
Mouse 6903
Log2 (tumor/kidney)
Position (Mb)
3.9
44.5
84.3
123.7
4
2
0
−2
−4

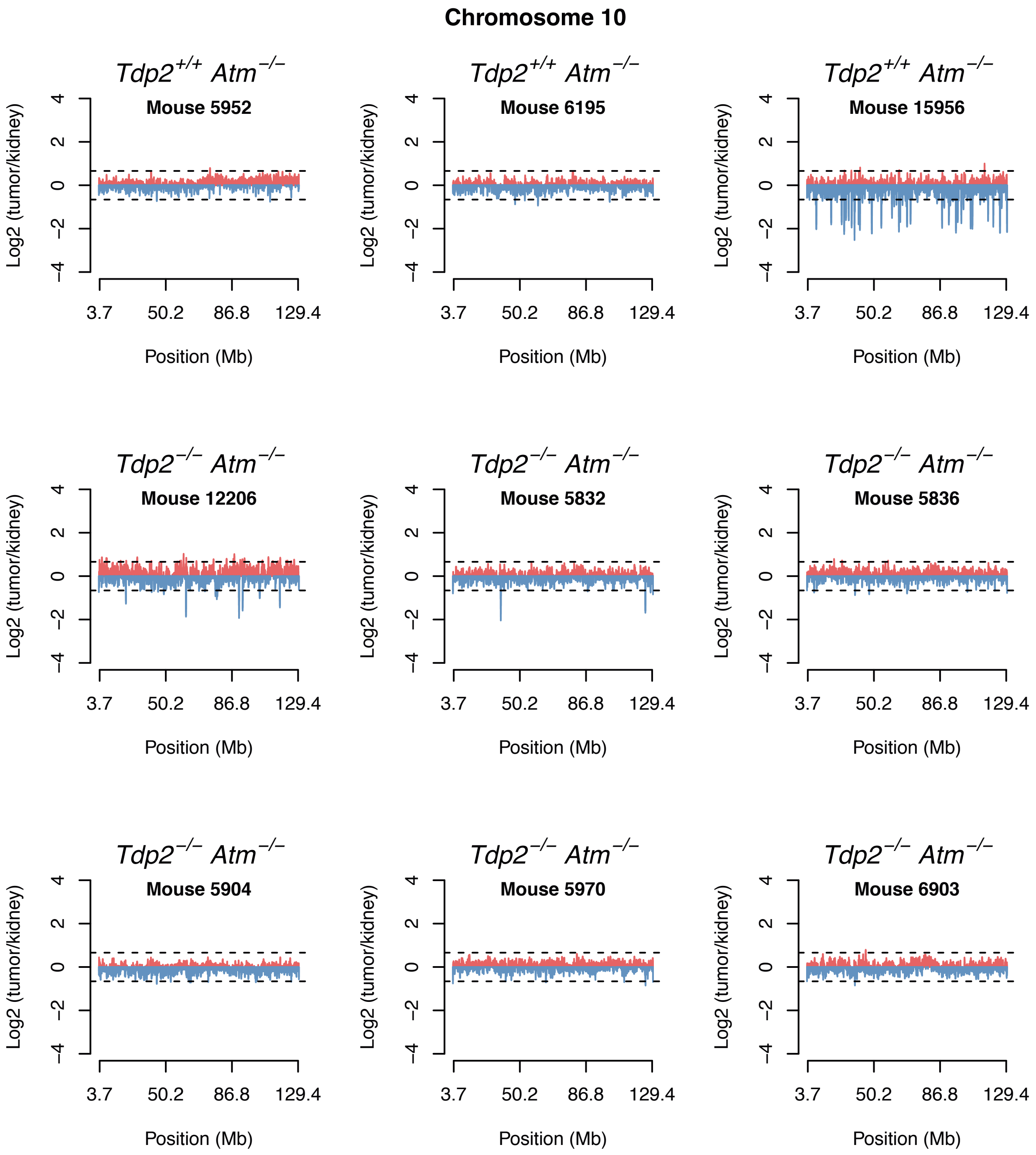
Chromosome 10
Tdp2+/+ Atm−/−
Mouse 5952
Tdp2+/+ Atm−/−
Mouse 6195
Tdp2+/+ Atm−/−
Mouse 15956
Tdp2−/− Atm−/−
Mouse 12206
Tdp2−/− Atm−/−
Mouse 5832
Tdp2−/− Atm−/−
Mouse 5836
Tdp2−/− Atm−/−
Mouse 5904
Tdp2−/− Atm−/−
Mouse 5970
Tdp2−/− Atm−/−
Mouse 6903
Log2 (tumor/kidney)
Position (Mb)
4
2
0
−2
−4
3.7
50.2
86.8
129.4

Chromosome 11

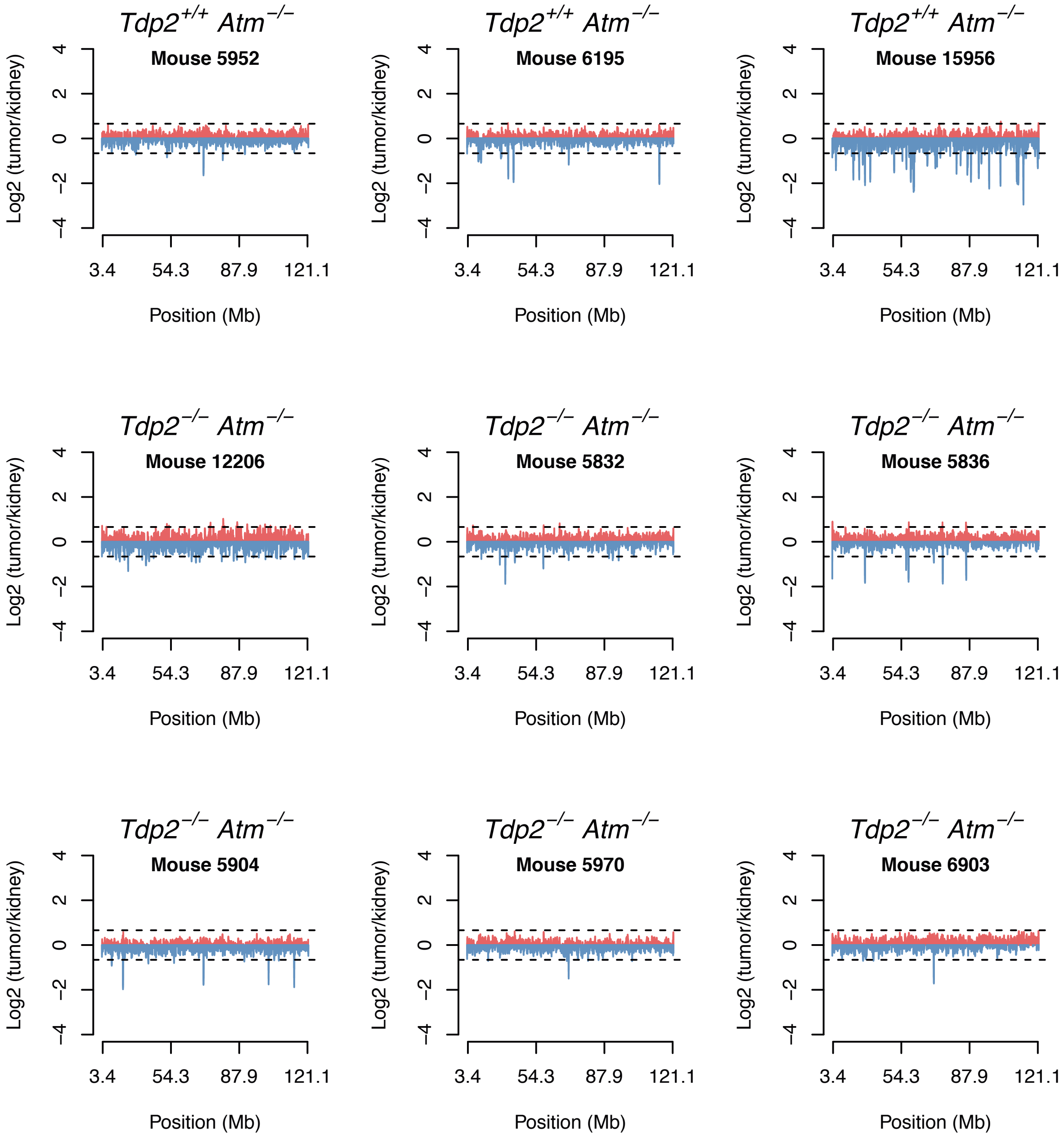

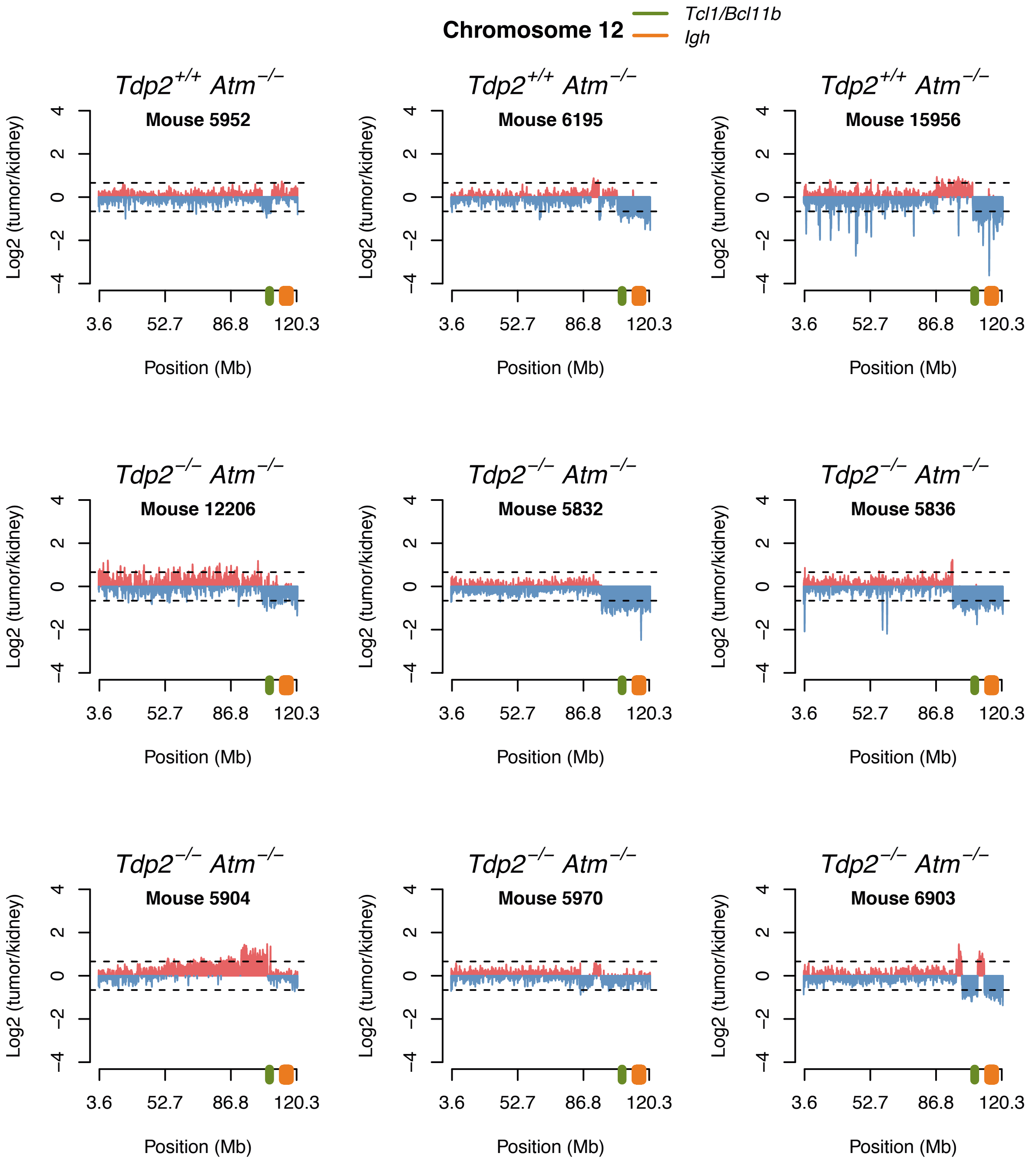
Chromosome 12
Tcl1/Bcl11b
Igh
Tdp2+/+ Atm−/−
Mouse 5952
Mouse 6195
Mouse 15956
Tdp2−/− Atm−/−
Mouse 12206
Mouse 5832
Mouse 5836
Mouse 5904
Mouse 5970
Mouse 6903
Log2 (tumor/kidney)
4
2
0
−2
−4
3.6
52.7
86.8
120.3
Position (Mb)

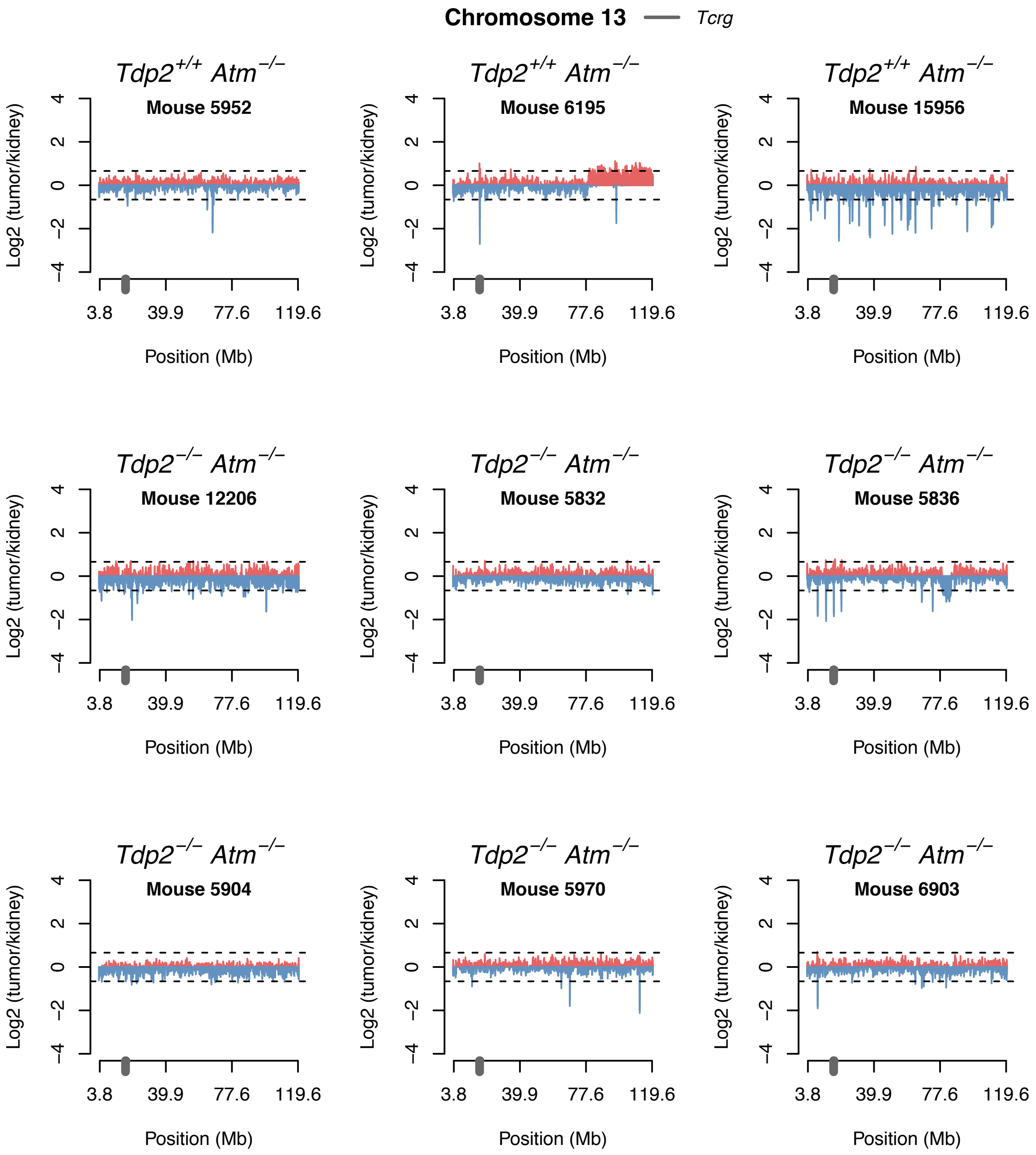
Chromosome 13
Tcrg
Tdp2+/+ Atm−/−
Mouse 5952
Tdp2+/+ Atm−/−
Mouse 6195
Tdp2+/+ Atm−/−
Mouse 15956
Tdp2−/− Atm−/−
Mouse 12206
Tdp2−/− Atm−/−
Mouse 5832
Tdp2−/− Atm−/−
Mouse 5836
Tdp2−/− Atm−/−
Mouse 5904
Tdp2−/− Atm−/−
Mouse 5970
Tdp2−/− Atm−/−
Mouse 6903
Log2 (tumor/kidney)
4
2
0
−2
−4
3.8
39.9
77.6
119.6
Position (Mb)

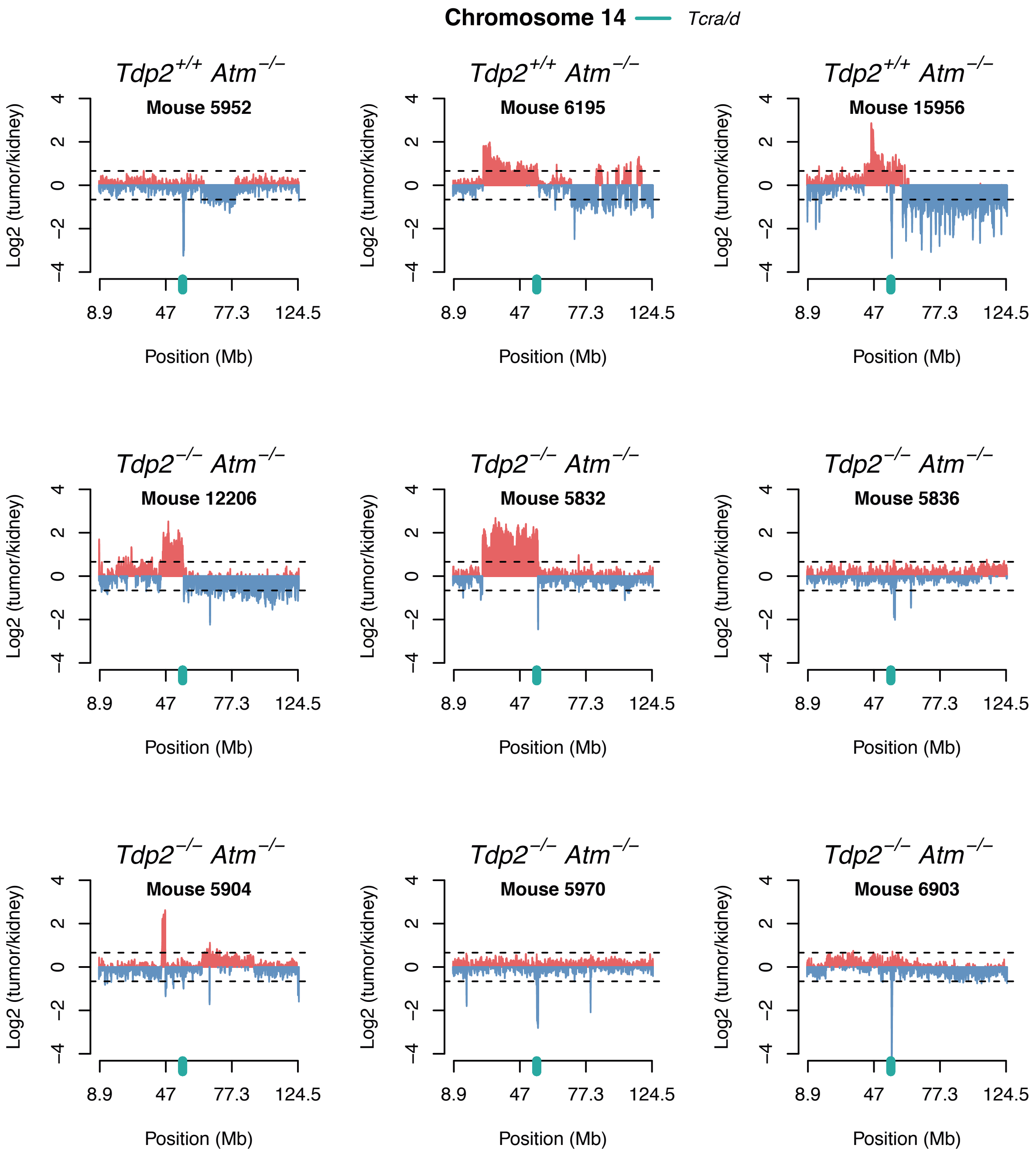

Chromosome 14
Tcra/d
Tdp2+/+ Atm−/−
Mouse 5952
Tdp2+/+ Atm−/−
Mouse 6195
Tdp2+/+ Atm−/−
Mouse 15956
Tdp2−/− Atm−/−
Mouse 12206
Tdp2−/− Atm−/−
Mouse 5832
Tdp2−/− Atm−/−
Mouse 5836
Tdp2−/− Atm−/−
Mouse 5904
Tdp2−/− Atm−/−
Mouse 5970
Tdp2−/− Atm−/−
Mouse 6903
Log2 (tumor/kidney)
−4 −2 0 2 4
8.9 47 77.3 124.5
Position (Mb)

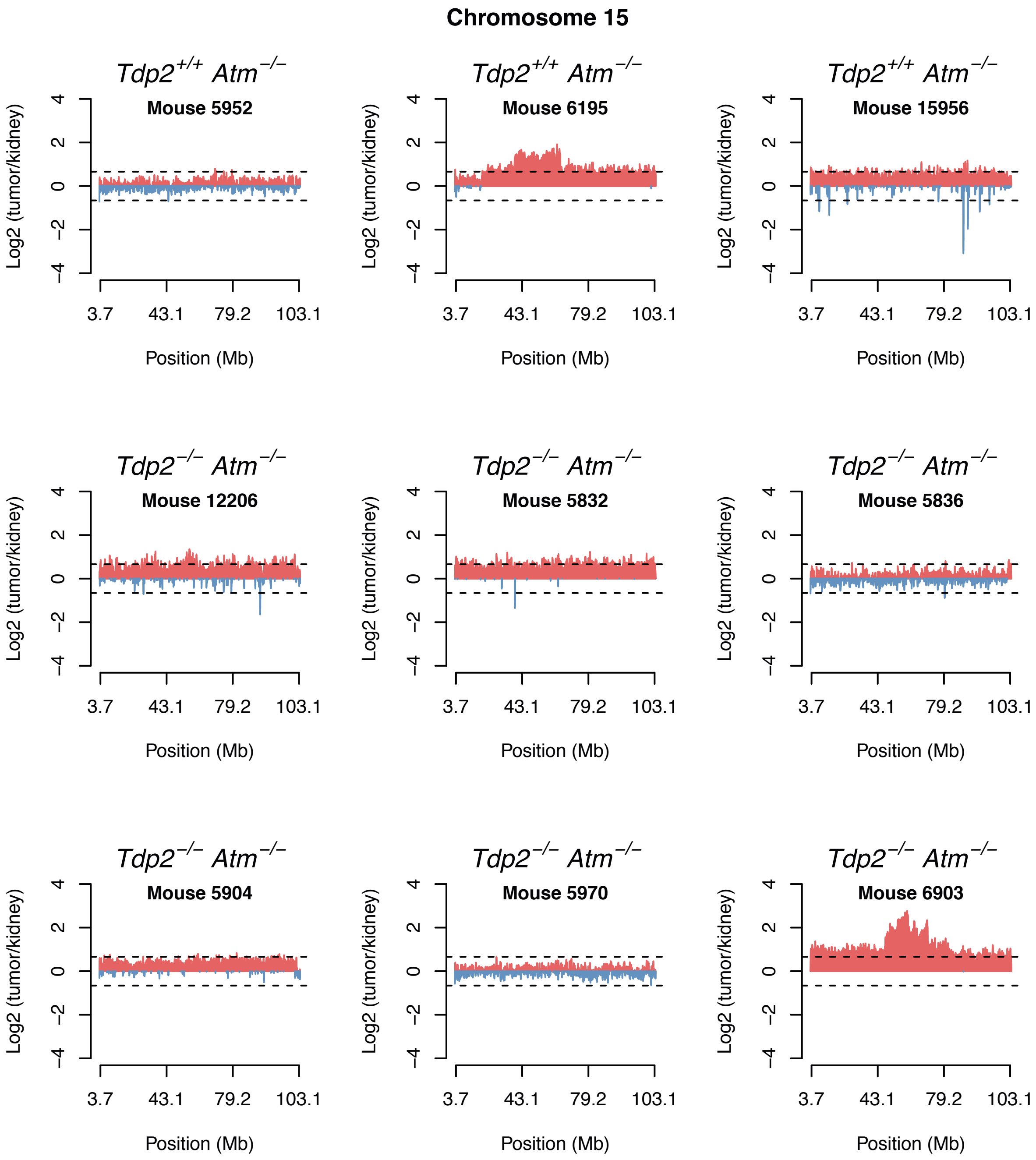
Chromosome 15
Tdp2+/+ Atm−/−
Mouse 5952
Tdp2+/+ Atm−/−
Mouse 6195
Tdp2+/+ Atm−/−
Mouse 15956
Tdp2−/− Atm−/−
Mouse 12206
Tdp2−/− Atm−/−
Mouse 5832
Tdp2−/− Atm−/−
Mouse 5836
Tdp2−/− Atm−/−
Mouse 5904
Tdp2−/− Atm−/−
Mouse 5970
Tdp2−/− Atm−/−
Mouse 6903
Log2 (tumor/kidney)
4
2
0
−2
−4
3.7
43.1
79.2
103.1
Position (Mb)

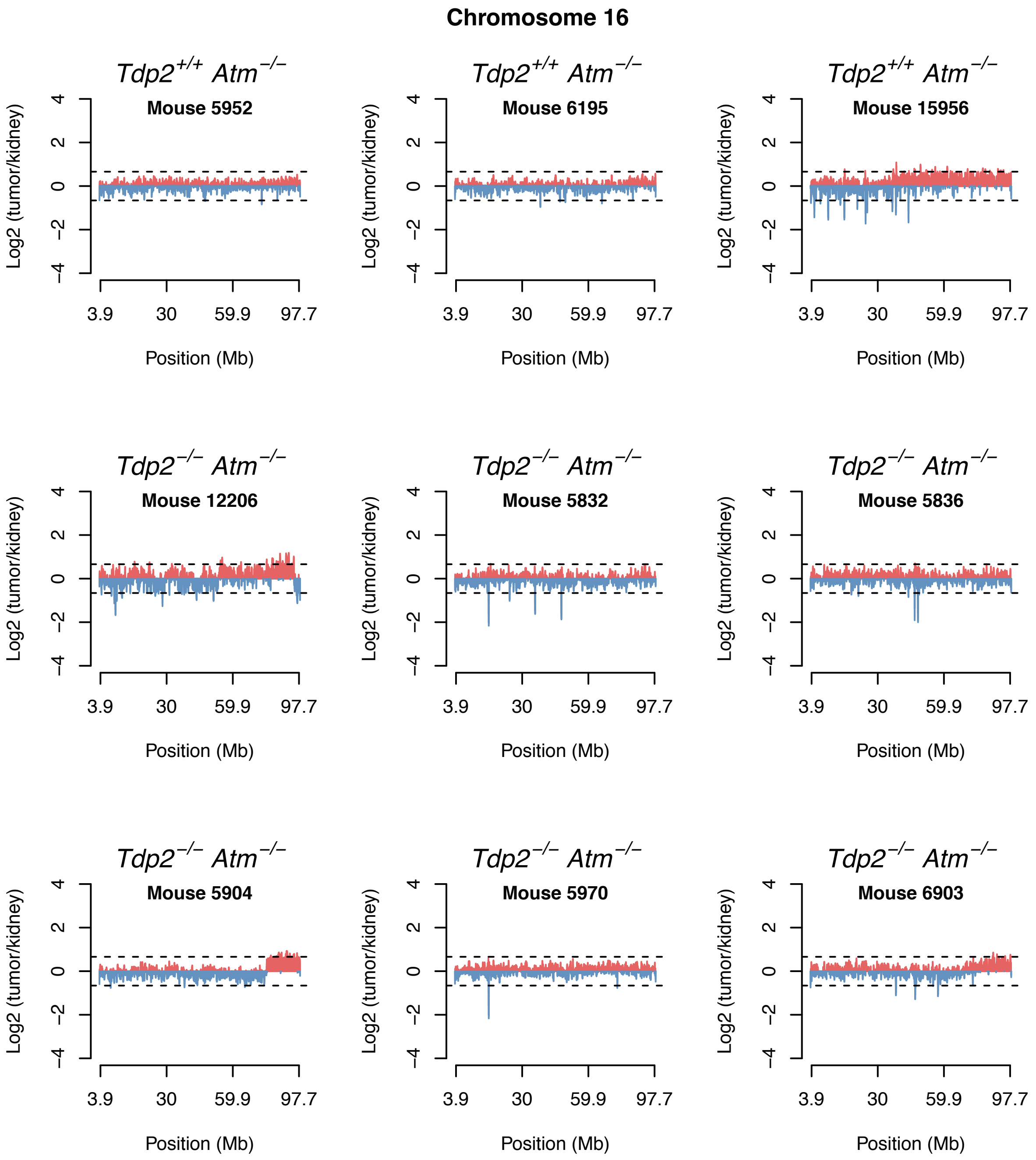
Chromosome 16
Tdp2+/+ Atm−/−
Mouse 5952
Tdp2+/+ Atm−/−
Mouse 6195
Tdp2+/+ Atm−/−
Mouse 15956
Tdp2−/− Atm−/−
Mouse 12206
Tdp2−/− Atm−/−
Mouse 5832
Tdp2−/− Atm−/−
Mouse 5836
Tdp2−/− Atm−/−
Mouse 5904
Tdp2−/− Atm−/−
Mouse 5970
Tdp2−/− Atm−/−
Mouse 6903
Log2 (tumor/kidney)
4
2
0
−2
−4
3.9
30
59.9
97.7
Position (Mb)

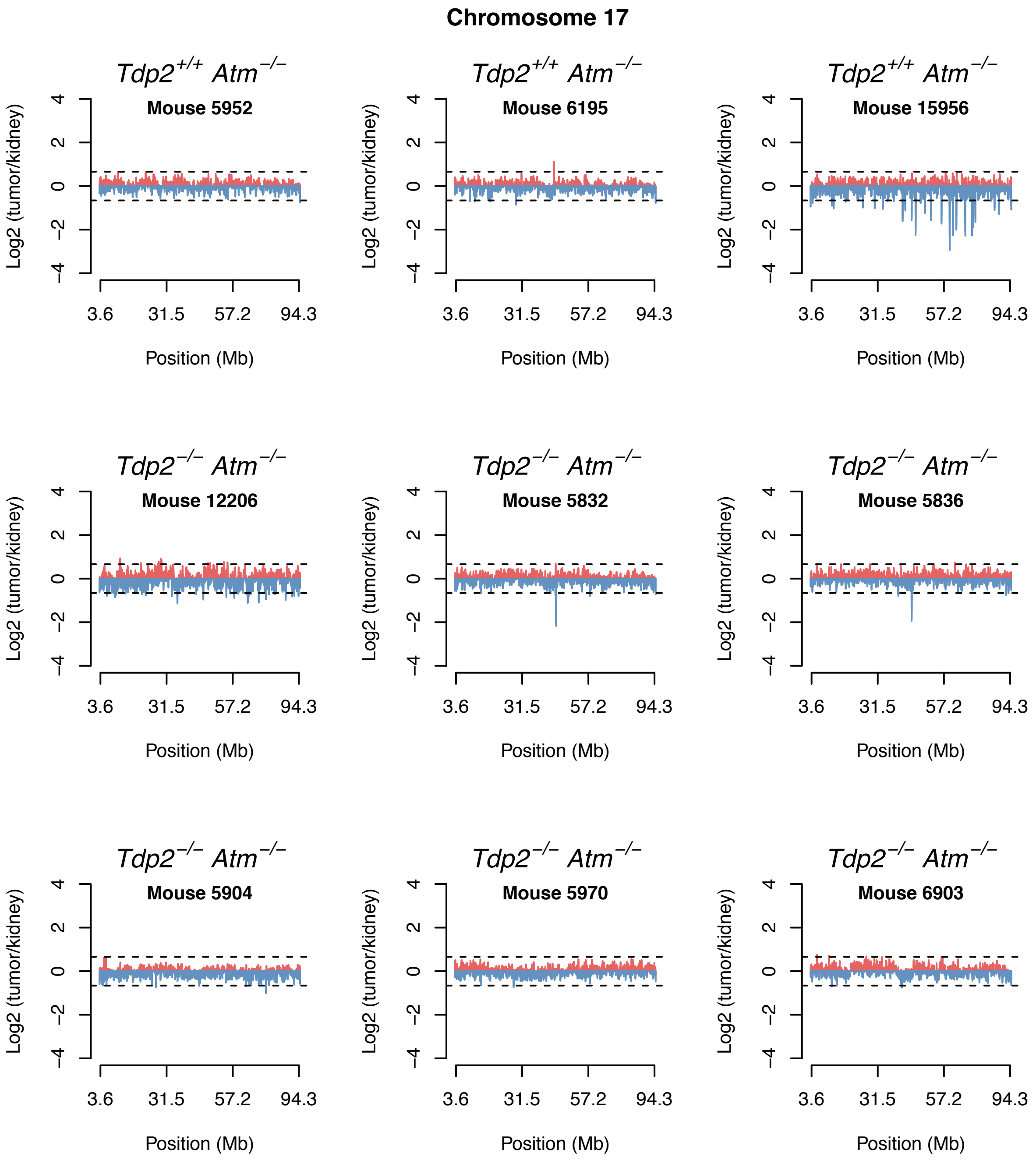
Chromosome 17
Tdp2+/+ Atm−/−
Mouse 5952
Tdp2+/+ Atm−/−
Mouse 6195
Tdp2+/+ Atm−/−
Mouse 15956
Tdp2−/− Atm−/−
Mouse 12206
Tdp2−/− Atm−/−
Mouse 5832
Tdp2−/− Atm−/−
Mouse 5836
Tdp2−/− Atm−/−
Mouse 5904
Tdp2−/− Atm−/−
Mouse 5970
Tdp2−/− Atm−/−
Mouse 6903
Log2 (tumor/kidney)
4
2
0
−2
−4
3.6
31.5
57.2
94.3
Position (Mb)

Chromosome 18

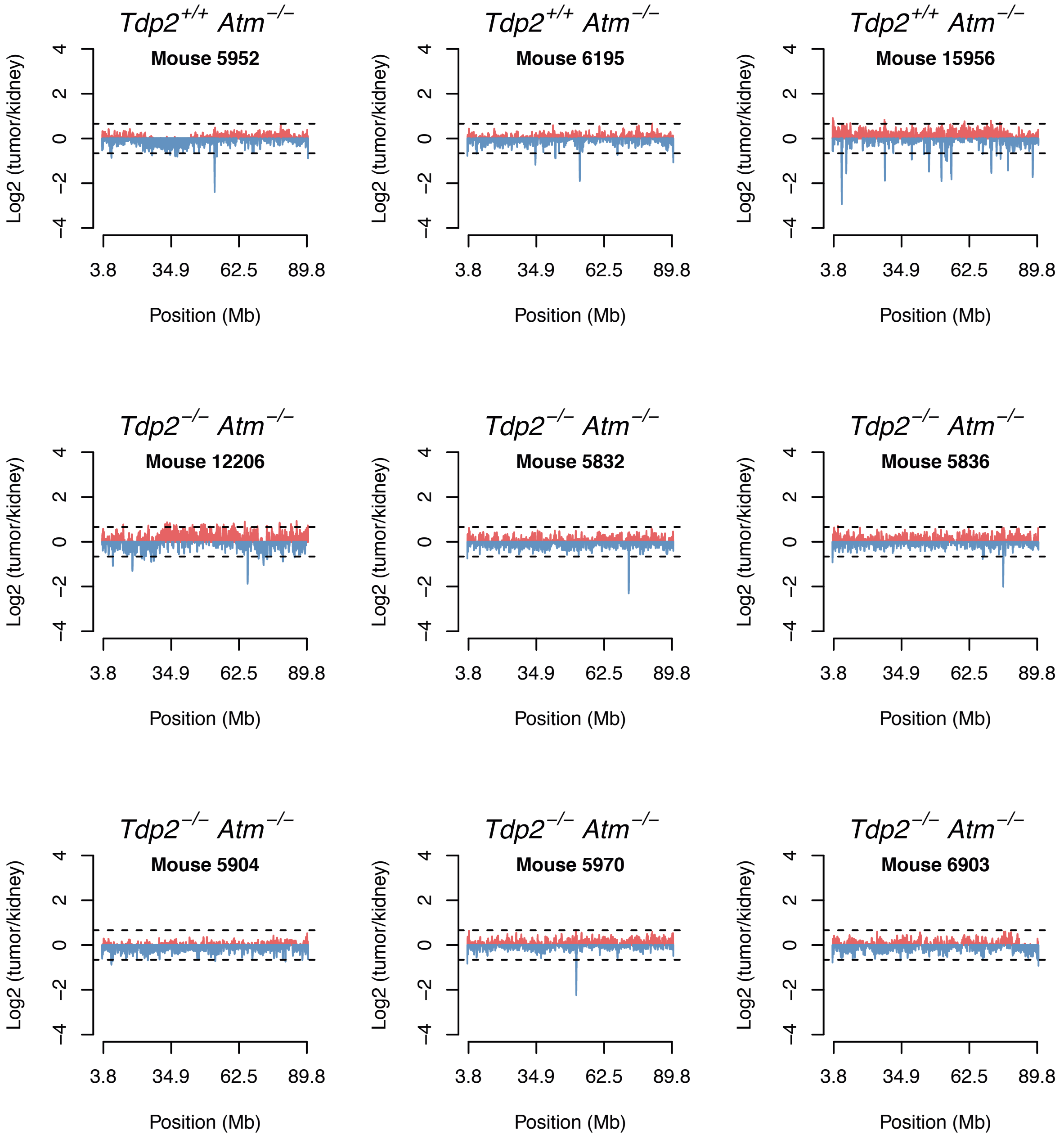

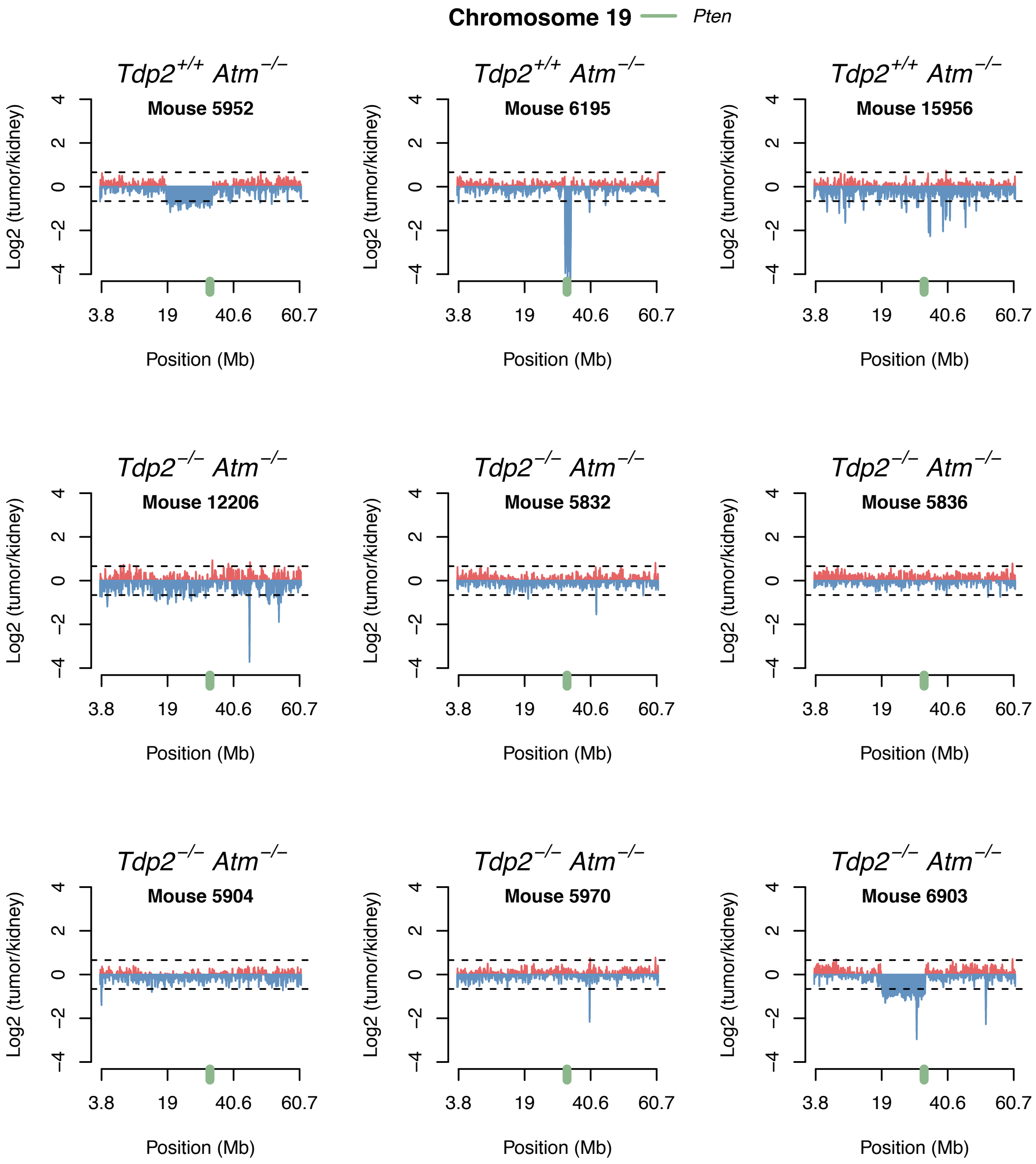
Chromosome 19
Pten
Tdp2+/+ Atm−/−
Mouse 5952
Mouse 6195
Mouse 15956
Tdp2−/− Atm−/−
Mouse 12206
Mouse 5832
Mouse 5836
Mouse 5904
Mouse 5970
Mouse 6903
Log2 (tumor/kidney)
Position (Mb)
3.8
19
40.6
60.7
4
2
0
−2
−4

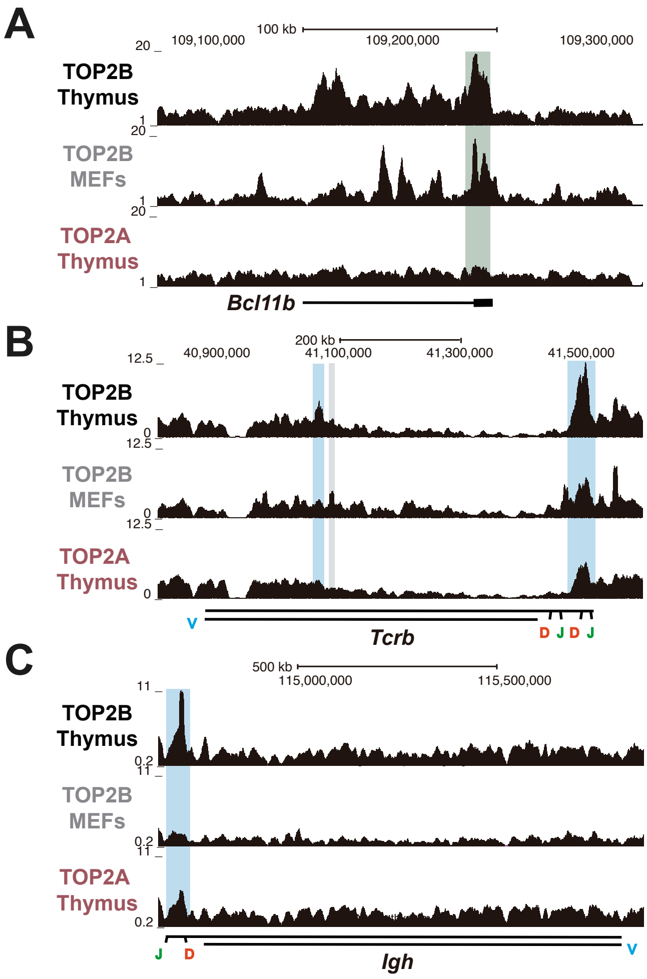

Supplementary Figure 4. TOP2 enrichment is preferential for thymocytes and the TOP2B isoform. (A-C) Comparison of TOP2B and TOP2A binding in thymocytes and MEFs. Genome browser view of TOP2B and TOP2A signal tracks in thymocytes and TOP2B signal in MEFs at *Bcl11b* **(A)**, *Tcrb* **(B)** and *IgH* **(C)** loci. TOP2B peaks unique in thymocytes (blue) or MEFs (grey), or shared in both cell types (green) are highlighted. No TOP2A peaks were detected in these domains.

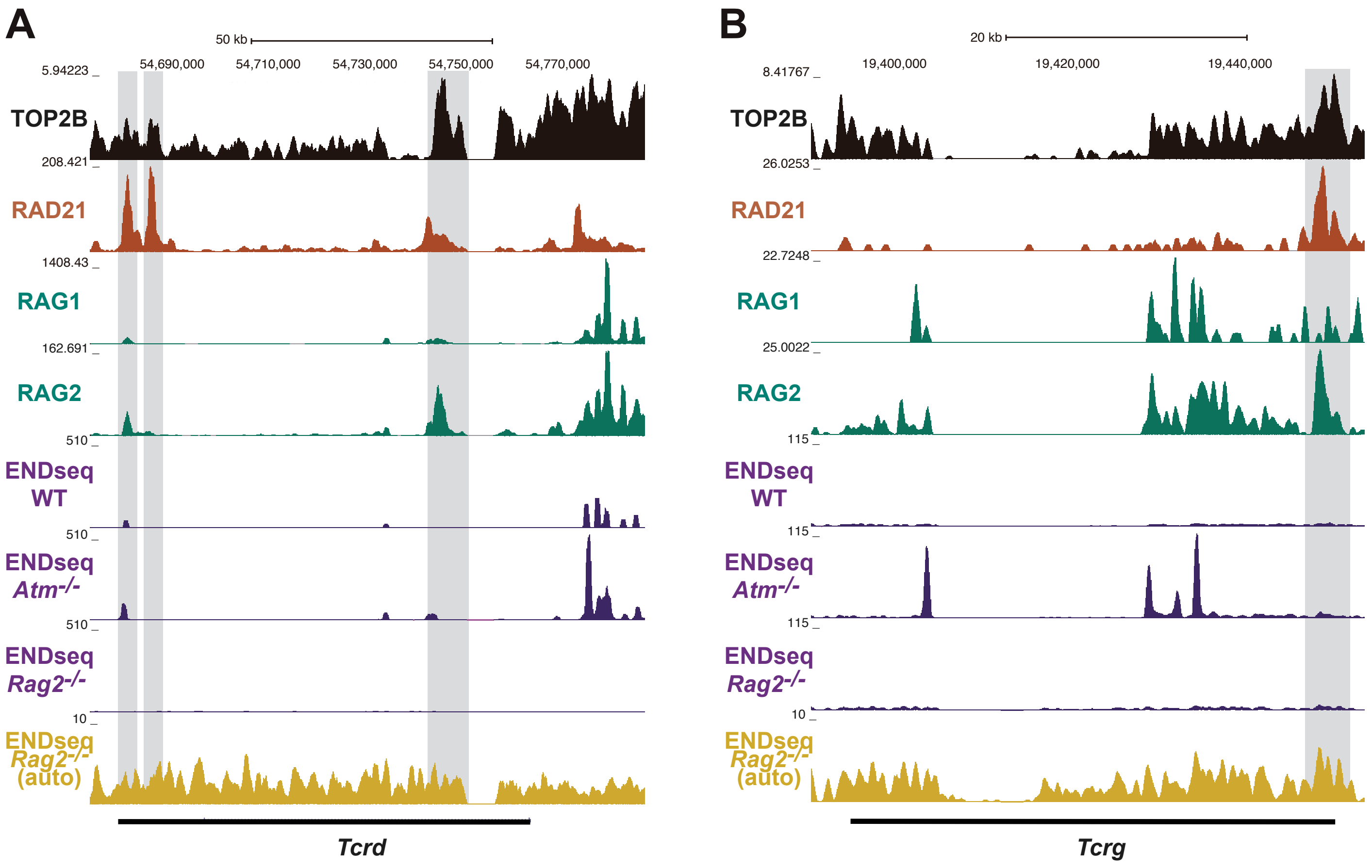

Supplementary Figure 5. TOP2B enrichment at additional V(D)J-active regions. (A,B) Genome browser view of TOP2B, RAD21, RAG1, RAG2 and ENDseq signal tracks in wild-type, *Atm*$^{-/-}$ and *Rag2*$^{-/-}$ primary thymocytes, as indicated, at *Tcrd* **(A)** and *Tcrg* **(B)** loci. TOP2B enriched regions are highlighted in grey. ENDseq signal in *Rag2*$^{-/-}$ thymocytes is additionally shown with a smaller scale to appreciate sites of minor DSB accumulation (yellow, auto).

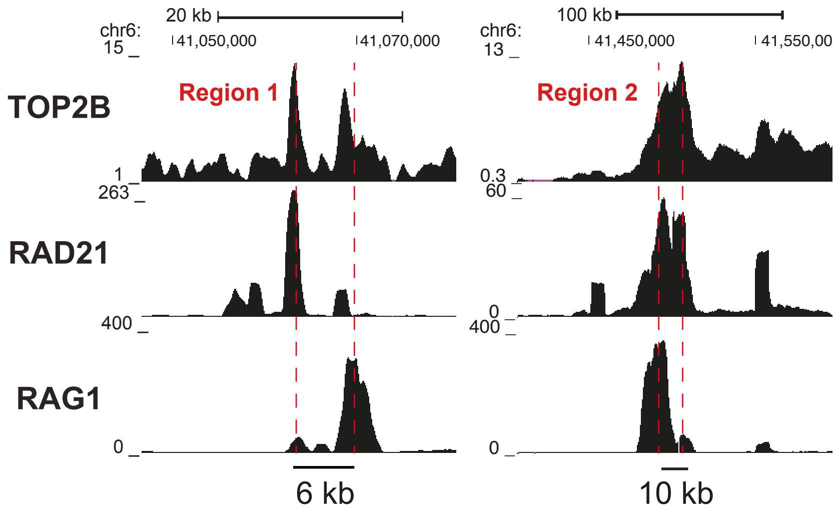

Supplementary Figure 6. Displacement between RAG1 and TOP2B binding at *Tcrb* sites. Zoom-in at regions 1 and 2 (Figure 7C) of TOP2B binding at the *Tcrb* locus. Distance between RAG1 and nearby TOP2B peaks is indicated (bottom).

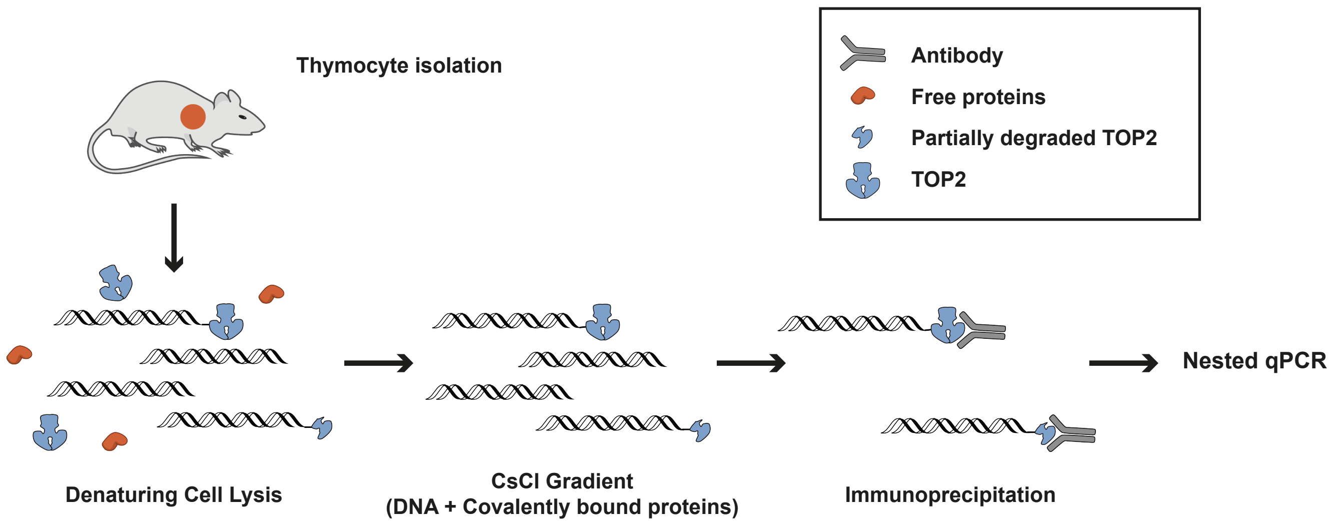

Supplementary Figure 7. Schematic overview of the ICE-IP method. Freshly isolated thymocytes are lysed under denaturing conditions and subjected to CsCl centrifugation to isolate nucleic acids and covalently attached proteins. Material is immunoprecipitated with TOP2B-specific antibodies and used for nested qPCR detection of specific genomic regions.

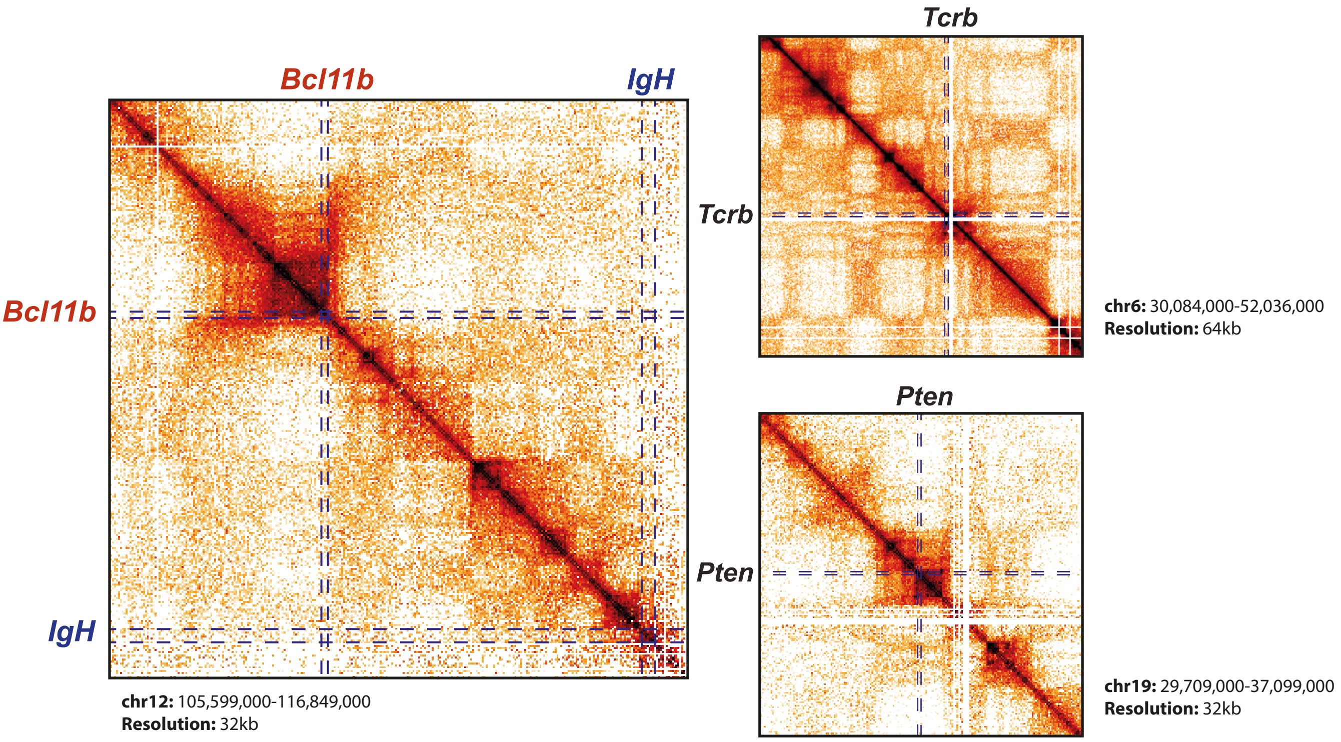

Supplementary Figure 8. *Atm*$^{-/-}$ dependent translocation hotspots and 3D genome architecture. Contact matrixes from wild-type thymocytes for regions of chromosome 12 (*Bcl11b and IgH*), chromosome 6 (*Tcrb*) and chromosome 19 (*Pten*) visualized using HiGlass web browser. Discontinuous lines delimit gene regions. Chromosome regions and resolution of matrices are shown at the bottom of each box.

Genotype	Mouse ID	Amp (*Tcra/d*)	Del (12)	Del (*Tcrb*)	Amp (15)	Del (*Pten*)	Amp (*Notch1*)	Other
Tdp2$^{+/+}$*Atm*$^{-/-}$	5952	-	+	-	-	+	-	Del(8)(18)
Tdp2$^{+/+}$*Atm*$^{-/-}$	6195	+	+	-	+	+	-	Amp(4)(13), Del(8)
Tdp2$^{+/+}$*Atm*$^{-/-}$	15956	+	+	+	+	-	-	Amp(16)
Tdp2$^{-/-}$ *Atm*$^{-/-}$	12206	+	+	+	+	-	-	Amp(1)(5)(16)(18)
Tdp2$^{-/-}$ *Atm*$^{-/-}$	5832	+	+	-	+	-	+	Amp(1)(4)(7)
Tdp2$^{-/-}$ *Atm*$^{-/-}$	5836	+	+	+	-	-	-	Del(4)(13)
Tdp2$^{-/-}$ *Atm*$^{-/-}$	5904	+	+	-	+	-	-	Amp(4)(16), Del(8)
Tdp2$^{-/-}$ *Atm*$^{-/-}$	5970	-	+	-	-	-	-	Amp(1)
Tdp2$^{-/-}$ *Atm*$^{-/-}$	6903	-	+	-	+	+	-	Amp(5)(16)

Supplementary Table 1. Genomic characterization of thymic tumours in *Atm*$^{-/-}$ and *Tdp2*$^{-/-}$ *Atm*$^{-/-}$ mice. Table summarising CGH analysis in each mouse identified by its ID number. Copy number variations (+) or not (-) at each locus of interest is indicated. Amp(*Tcra/d*): amplification upstream of *Tcra/d*; Del(12): deletion of the telomeric region of chromosome 12 covering *Bcl11b*; Del(*Tcrb*): deletion at the *Tcrb* locus; Amp(15): trisomy of chromosome 15; Del(*Pten*): deletion at the *Pten* locus; Amp(*Notch1*): duplication at the *Notch1* locus. Additional regions of amplification (Amp) or deletion (Del) in the indicated chromosome are summarised.
