# Supplementary material for: Endogenous topoisomerase II-mediated DNA breaks drive thymic cancer predisposition linked to ATM deficiency
Source: Nat Commun. 2020 Feb 14;11:910. doi: 10.1038/s41467-020-14638-w (PMC7021672; doi:10.1038/s41467-020-14638-w)
Supplement: Supplementary file 3 — Reporting Summary [file 41467_2020_14638_MOESM3_ESM.pdf]

nature research

Corresponding author(s): Felipe Cortes Ledesma

Last updated by author(s): Oct 18, 2019

Reporting Summary

Nature Research wishes to improve the reproducibility of the work that we publish. This form provides structure for consistency and transparency in reporting. For further information on Nature Research policies, see Authors & Referees and the Editorial Policy Checklist .

Statistics

For all statistical analyses, confirm that the following items are present in the figure legend, table legend, main text, or Methods section.

n/a	Confirmed	
☐	☒	The exact sample size (*n*) for each experimental group/condition, given as a discrete number and unit of measurement
☐	☒	A statement on whether measurements were taken from distinct samples or whether the same sample was measured repeatedly
☐	☒	The statistical test(s) used AND whether they are one- or two-sided<br>*Only common tests should be described solely by name; describe more complex techniques in the Methods section.*
☐	☒	A description of all covariates tested
☐	☒	A description of any assumptions or corrections, such as tests of normality and adjustment for multiple comparisons
☐	☒	A full description of the statistical parameters including central tendency (e.g. means) or other basic estimates (e.g. regression coefficient) AND variation (e.g. standard deviation) or associated estimates of uncertainty (e.g. confidence intervals)
☐	☒	For null hypothesis testing, the test statistic (e.g. *F*, *t*, *r*) with confidence intervals, effect sizes, degrees of freedom and *P* value noted<br>*Give P values as exact values whenever suitable.*
☒	☐	For Bayesian analysis, information on the choice of priors and Markov chain Monte Carlo settings
☒	☐	For hierarchical and complex designs, identification of the appropriate level for tests and full reporting of outcomes
☒	☐	Estimates of effect sizes (e.g. Cohen's *d*, Pearson's *r*), indicating how they were calculated

Our web collection on statistics for biologists contains articles on many of the points above.

Software and code

Policy information about availability of computer code

Data collection	NGS data used were collected from GEO and ENCODE repository and no specific software was used for this purpose.
Data analysis	UCSC Genome Browser – Kent et al., 2002. http://genome.ucsc.edu<br>GraphPad Prism v6. - https://graphpad.com<br>MACS - Zhang et al. 2008. https://pypi.python.org/pypi/MACS<br>Bowtie 1.2.0. (Mapping) – Langmead et al. 2009. https://sourceforge.net/projects/bowtie-bio/files/bowtie<br>HOMER (Peak calling) – Heinz et al. 2010. https://homer.ucsd.edu/homer<br>SAMTOOLS-1.1 – Li et al., 2009. https://github.com/samtools/samtools<br>deepTools-2.4.1. – Ramirez et al., 2016. https://deeptools.readthedocs.io<br>R – R Development Core Team, 2008. https//www.r-projects.org<br>SeqPlots – Stempor et al. 2016. https://github.com/Przemol/seqplots<br>HiGlass - Kerpedjiev et al. 2018. https://higlass.io/<br>FastQ Toolkit - http://hannonlab.cshl.edu/fastx_toolkit/<br>Trimmomatic - Bolger et al. 2014. doi:10.1093/bioinformatics/btu170<br>BD CellQuest pro. - https://www.bd.com/<br>FlowJo v9. - https://www.flowjo.com/solutions/flowjo

For manuscripts utilizing custom algorithms or software that are central to the research but not yet described in published literature, software must be made available to editors/reviewers. We strongly encourage code deposition in a community repository (e.g. GitHub). See the Nature Research guidelines for submitting code & software for further information.

Data

Policy information about availability of data

All manuscripts must include a data availability statement. This statement should provide the following information, where applicable:

- Accession codes, unique identifiers, or web links for publicly available datasets
- A list of figures that have associated raw data
- A description of any restrictions on data availability

The authors confirm that the data supporting the findings of this study are available within the article [and/or] its supplementary materials. The raw data is available from the corresponding author upon request.

Next-generation sequencing data are available at the Gene Expression Omnibus (GEO) database under accession number GSE133954 [https://www.ncbi.nlm.nih.gov/geo/query/acc.cgi?acc=GSE133954]. CGH arrays have been deposited at the GEO database under accession number GSE134054 [https://www.ncbi.nlm.nih.gov/geo/query/acc.cgi?acc=GSE134054].

Other available datasets used in this study:

RAG1 and RAG2 ChIPseq in thymocytes - SRA: PRJNA285688. Thymocytes ENDSeq - SRA: PRJNA326246. RAD21 ChIPseq in DP T-cells - SRA: PRJNA432324. POLR2A, H4K4me3, H3K27ac and CTCF – ENCODE: ENCSR000CEA, ENCSR000CCJ, ENCSR000CCH and ENCSR000CDZ respectively. Hi-C data in thymocytes - SRA: PRJNA435621.

Field-specific reporting

Please select the one below that is the best fit for your research. If you are not sure, read the appropriate sections before making your selection.

☒ Life sciences ☐ Behavioural & social sciences ☐ Ecological, evolutionary & environmental sciences

For a reference copy of the document with all sections, see nature.com/documents/nr-reporting-summary-flat.pdf

Life sciences study design

All studies must disclose on these points even when the disclosure is negative.

Sample size	Sample size was determined according to the minimal number of independent biological replicates that significantly identified an effect.
Data exclusions	No data were excluded.
Replication	All experiments in this study were independently replicated, with biological and technical replicates as indicated in Figure Legends.
Randomization	Animals were asigned an individual identification number (ID) upon birth. Animals of each genotype were randomly allocated to specific treatment conditions only considering their ID.
Blinding	Genotype and treatment were codified in an excel file for each animal ID. Throughout experimental collection of the data, researchers only had access to the animal ID.

Reporting for specific materials, systems and methods

We require information from authors about some types of materials, experimental systems and methods used in many studies. Here, indicate whether each material, system or method listed is relevant to your study. If you are not sure if a list item applies to your research, read the appropriate section before selecting a response.

Materials & experimental systems

n/a	Involved in the study	
☐	☒	Antibodies
☐	☒	Eukaryotic cell lines
☒	☐	Palaeontology
☐	☒	Animals and other organisms
☒	☐	Human research participants
☒	☐	Clinical data

Methods

n/a	Involved in the study	
☐	☒	ChIP-seq
☐	☒	Flow cytometry
☒	☐	MRI-based neuroimaging

Antibodies

Antibodies used	Anti-TOP2B, rabbit polyclonal – Novus Biologicals – NB100-40842

Anti-TOP2B, rabbit polyclonal – SantaCruz – sc13059
Anti-TOP2A, rabbit polyclonal - Abcam - ab52934
IgG rabbit – SIGMA - I8140
Anti-Mouse CD4 FITC, mouse monoclonal (Clone: RM4-4) – eBioscience – 11-0043-81
Anti-Mouse CD8a APC, mouse monoclonal (Clone: 53-6.7) – eBioscience – 17-0081-81
Anti-53BP1, rabbit polyclonal – SantaCruz – sc22760
B220-APC - eBioscience- 17-0452
CD43-FITC - eBioscience- 11-0431
Anti-calbindin - Swant - CB-38a
Goat Anti-rabbit secondary antibody - Jackson ImmunoResearch - 111-065-003
AlexaFluor conjugated secondary antibody - Jackson ImmunoResearch - 111-545-144

Validation

All antibodies validated by manufacturer.

Citations.

Anti-TOP2B (Novus Biologicals) .
Hedayati M et al. Clin Cancer Res 2016. PMID: 26831716.
Maxwell SS et al. RNA Biol 2013. PMID: 24270455.

AntiTOP2B (SantaCruz).
Canela et al. Cell 2017. PMID: 28735753.
Uusküla et al. Genome Biol 2016. PMID: 27582050.

Anti-Mouse CD4 FITC.
Downs-Canner et al. Nat Comm 2017. PMID: 28290453.
Szalay et al. Nat Comm 2016. PMID: 27139776.

Anti-Mouse CD8a APC.
Ng et al. Elife 2018. PMID: 30457103.
Ravussin et al. Cell Rep 2018. PMID: 30067966.

Anti-TOP2A (Abcam).
Nicholls et al. Mol Cell. 2018. PMID: 29290614.
Canela et al. Cell. 2017. PMID: 28735753.

Anti-53BP1 (SantaCruz).
Burrell et al. Nature 2013. PMID: 23446422.
Beck et al. Mol Cell. 2012. PMID: 22907750.

B220-APC - eBioscience- 17-0452.
Advanced Verification by ThermoFisher.
Glal et al. Front Immunol 2018. PMID: 30455690.
Liu et al. Front Immunol 2018. PMID: 29623080.

CD43-FITC - eBioscience- 11-0431.
Wang et al. J Bone Miner Res. 2018. PMID: 29206332.
Delás et al. Elife. 2017. PMID: 28875933.

Anti-calbindin - Swant - CB-38a.
Airaksinen et al. PNAS. 1997.

Goat Anti-rabbit secondary antibody - Jackson ImmunoResearch - 111-065-003.
Giguère et al. PLos Genetic. 2019. PMID: 31449520.
Real et al. 2019. PMID: 29271291

AlexaFluor conjugated secondary antibody - Jackson ImmunoResearch - 111-545-144.
Koike et al. Nature Communications. 2019. PMID: 30962439.
Zhu et al. Nature Communications. 2019. PMID: 30842412.

Eukaryotic cell lines

Policy information about cell lines

Cell line source(s)	Primary embryonic fibroblast and thymocytes were isolated from mice.
Authentication	n/a
Mycoplasma contamination	n/a
Commonly misidentified lines (See ICLAC register)	n/a

Animals and other organisms

Policy information about studies involving animals; ARRIVE guidelines recommended for reporting animal research

Laboratory animals

Wild-type, Tdp2-/-, Atm-/- and Tdp2-/-Atm-/- mice were generated by crossing Tdp2 +/- mice in mixed 129Ola x CD1 x C57BL/6 background and Atm+/- mice in mixed 129/SvEv x NIH Black Swiss background. Double heterozygous Tdp2+/- Atm+/-mice were selected and bred to maintain the colony and generate mice with the required genotypes.

Trp53-/- and Tdp2-/- Trp53-/- were generated by crossing Tdp2 +/- mice in mixed 129Ola, CD1 and C57BL/6 background and Trp53+/- mice in mixed 129/SvEv x C57BL/6 background. Double heterozygous Tdp2+/- Trp53+/- mice were selected and bred to maintain the colony and generate mice with the required genotypes.

Both male and females were used indistinctly.

For the analysis of lifespan and tumour incidence, animals were maintained throughout their life with a maximum of 2 years.

For individual treatment experiments, animals were used at 4 or 8 weeks of age, as indicated.

Wild animals	No wild animals were used in the study.
Field-collected samples	No field-collected samples were used in the study.
Ethics oversight	All animal procedures were performed in accordance with European Union legislation and with the approval of the local Committee of CABIMER and the Andalusian Regional Government (Junta de Andalucía, Consejería de Agricultura, Pesca y Desarrollo). Authorisation number 23/02/2018/14

Note that full information on the approval of the study protocol must also be provided in the manuscript.

ChIP-seq

Data deposition

- [x] Confirm that both raw and final processed data have been deposited in a public database such as GEO.
- [x] Confirm that you have deposited or provided access to graph files (e.g. BED files) for the called peaks.

Data access links
May remain private before publication.

https://www.ncbi.nlm.nih.gov/geo/query/acc.cgi?acc=GSE133954 (reviewer token: gnsraugulpsjlwx)
https://www.ncbi.nlm.nih.gov/geo/query/acc.cgi?&acc=GSE134054 (reviewer token: ynszkuguhrcvzqx)

Files in database submission

GSM3931373	TOP2B SC ChIPSeq
GSM3931374	TOP2B Novus ChIPSeq
GSM4121365	TOP2A ChIPSeq
GSM3931375	IgG ChIPSeq
GSM3934872	CGH Tdp2-/-Atm-/- Exp1
GSM3934873	CGH Tdp2-/-Atm-/- Exp2
GSM3934874	CGH Tdp2-/-Atm-/- Exp3
GSM3934875	CGH Tdp2-/-Atm-/- Exp4
GSM3934876	CGH Tdp2-/-Atm-/- Exp5
GSM3934877	CGH Tdp2-/-Atm-/- Exp6
GSM3934878	CGH Tdp2+/+Atm-/- Exp1
GSM3934879	CGH Tdp2+/+Atm-/- Exp2
GSM3934880	CGH Tdp2+/+Atm-/- Exp3

Genome browser session (e.g. UCSC)

http://genome.ucsc.edu/s/JoseTerron/nature_communication

Methodology

Replicates	Two replicates with two different antibodies
Sequencing depth	Single-end reads of 75 bp.<br>ChIPseq TOP2B (SantaCruz ab) -> 76 million reads. 64% uniquely mapped.<br>ChIPseq TOP2B (NB) -> 62 million reads. 65% uniquely mapped.<br>ChIPseq TOP2A -> 28 million reads. 75% uniquely mapped.<br>ChIPseq IgG -> 66 millions reads. 78% uniquely mapped.
Antibodies	Anti-TOP2B, rabbit polyclonal – Novus Biologicals – NB100-40842<br>Anti-TOP2B, rabbit polyclonal – SantaCruz – sc13059<br>Anti-TOP2A, rabbit polyclonal - Abcam - ab52934<br>IgG rabbit – SIGMA - I8140
Peak calling parameters	Peak calling using HOMER using standard parameters and factor style.
Data quality	Initial fitering and trimming was performed using FASTQ Toolkit v1.0.0.<br>Second trimming was performed using Trimmomatic (20:4 sliding window, 2:leading, 2:trailing, 35:minlen).<br><br>All peaks above 10-fold enrichment above control (IgH ChIPseq) and at FDR 1%.
Software	FASTQ Toolkit v1.0.0. from Illumina<br>Trimmomatic 0.36 - Bolger et al. 2014. http://www.usadellab.org/cms/?page=trimmomatic<br>Bowtie 1.2.0. (Mapping) – Langmead et al. 2009. https://sourceforge.net/projects/bowtie-bio/files/bowtie<br>HOMER (Peak calling) – Heinz et al. 2010. https://homer.ucsd.edu/homer<br>SAMTOOLS-1.1 – Li et al., 2009. https://github.com/samtools/samtools<br>deepTools-2.4.1. (coverage files) – Ramirez et al., 2016. Hhttps://deeptools.readthedocs.io<br>UCSC Genome Browser – Kent et al., 2002. http://genome.ucsc.edu<br>R – R Development Core Team, 2008. https//www.r-projects.org

Flow Cytometry

Plots

Confirm that:

- [x] The axis labels state the marker and fluorochrome used (e.g. CD4-FITC).
- [x] The axis scales are clearly visible. Include numbers along axes only for bottom left plot of group (a 'group' is an analysis of identical markers).
- [x] All plots are contour plots with outliers or pseudocolor plots.
- [x] A numerical value for number of cells or percentage (with statistics) is provided.

Methodology

Sample preparation	Healthy thymus (4 weeks old) or thymic lymphomas were extracted from mice, disaggregated to single cells in EDTA-buffer and immunostained as described in materials and methods.
Instrument	BD FACSCalibur Flow Cytometer (BD Biosciences)
Software	BD CellQuest Pro software
Cell population abundance	Total single cells from thymus/thymic lymphomas were used.
Gating strategy	Main population of single cells in SSC/FSC (>95% of events) were gated for analysis as described.

- [x] Tick this box to confirm that a figure exemplifying the gating strategy is provided in the Supplementary Information.
